# Establishment and evaluation of a novel practical tool for the screening of metabolic dysfunction-associated steatotic liver disease in patients with type 2 diabetes mellitus

**DOI:** 10.3389/fnut.2025.1692394

**Published:** 2025-12-12

**Authors:** Mingkang Zhang, Yazhi Wang, Meng Liu, Qi Shi, Xin'an Wu, Yan Zhou

**Affiliations:** 1 Department of Pharmacy, The First Hospital of Lanzhou University, Lanzhou, China; 2 School of Pharmacy, Lanzhou University, Lanzhou, China; 3 The Second School of Clinical Medicine, Lanzhou University, Lanzhou, China; 4 Department of Pharmacy, The Third Affiliated Hospital of Xi'an Medical College, Xi'an, China; 5 Suzhou Medical College, Soochow University, Suzhou, China

**Keywords:** type 2 diabetes mellitus, metabolic dysfunction-associated steatotic liver disease, diagnostic predictive nomogram, NHANES database, clinical practice

## Abstract

**Background:**

Metabolic dysfunction-associated steatotic liver disease (MASLD) is a major comorbidity in type 2 diabetes mellitus (T2DM), yet early prediction models tailored to this population are limited. This study aimed to develop and validate a novel diagnostic predictive model for MASLD in adults with T2DM.

**Methods:**

A total of 4,726 T2DM patients were retrospectively analyzed. Candidate predictors were screened by least absolute shrinkage and selection operator (LASSO) regression, and a multivariable logistic regression model was built. Significant variables were integrated into a diagnostic predictive nomogram (DPN), with online and Excel-based calculators developed. Model performance was comprehensively evaluated and compared with four established models for fatty liver disease across training, internal, and external (NHANES) validation datasets. Subgroup analyses assessed generalizability.

**Results:**

Eight independent predictors (sex, age, body mass index, alanine aminotransferase, albumin, diabetes duration, triglycerides, and high-density lipoprotein cholesterol) were included in the final model. The DPN achieved robust discrimination in training set (AUC: 0.775, 95% CI: 0.759–0.791), validation set (0.767, 95% CI: 0.742–0.791), and test set (0.794, 95% CI: 0.749–0.839) compared to existing models. NRI and IDI confirmed improved predictive capacity (*P* < 0.05). Calibration curves were excellent in the training (*P* = 0.936, Brier score = 0.184), validation (*P* = 0.956, Brier score = 0.189), and test sets (*P* = 0.687, Brier score = 0.156). DCA and CIC further demonstrated higher clinical net benefit. Subgroup analyses confirmed stability and broad applicability.

**Conclusions:**

The DPN is a clinically practical and resource-efficient screening tool that enables early risk stratification for MASLD in patients with T2DM. Its implementation could streamline screening pathways and facilitate timely intervention in routine clinical practice.

## 1 Introduction

Diabetes mellitus is a heterogeneous metabolic disorder characterized by impairments in both glucose and lipid metabolism. It has emerged as the fastest-growing global health burden, with the number of affected individuals continuing to rise. According to the latest report from the International Diabetes Federation (IDF), the global number of adults living with diabetes is projected to reach 643 million by 2030 and 784 million by 2045 (1). Currently, over 90% of diabetes cases are attributed to type 2 diabetes mellitus (T2DM), a condition primarily driven by varying degrees of pancreatic β-cell dysfunction and insulin resistance (IR) (2). Despite ongoing advances in the therapeutic landscape, T2DM remains associated with a substantial burden of complications and comorbidities. Epidemiological data indicate that the prevalence of end-stage renal disease (ESRD), diabetic retinopathy (DR), cardiovascular disease (CVD), and chronic liver disease among individuals with diabetes ranges from 12%−55%, 2.6%, 40%, and 12.3%−57%, respectively (3).

Metabolic dysfunction-associated steatotic liver disease (MASLD), the term recently adopted to replace non-alcoholic fatty liver disease (NAFLD), is now encompassed under the umbrella of steatotic liver disease (SLD). This nomenclature shift, driven by a 2023 international consensus, aims to reduce stigma and improve disease awareness (4). The pathophysiological mechanisms linking T2DM and MASLD are multifactorial and intertwined, primarily driven by IR (5). In the diabetic state, hyperinsulinaemia coupled with IR enhances lipolysis, leading to elevated circulating free fatty acids (FFAs). These FFAs are taken up by the liver, promoting hepatic triglyceride accumulation. Simultaneously, dysregulation of key transcription factors like sterol regulatory element-binding protein 1c (SREBP-1c) exacerbates *de novo* lipogenesis. This lipid overload induces endoplasmic reticulum stress (ERS) and mitochondrial dysfunction, resulting in increased reactive oxygen species (ROS) production, impaired β-oxidation, and heightened inflammation (6).

Furthermore, the coexistence of MASLD has been shown to exert a deleterious effect on the long-term prognosis and survival of individuals with T2DM. For example, individuals without diabetes who present with MASLD at baseline exhibit a significantly increased risk of developing T2DM over time, even after adjusting for conventional diabetes-related risk factors (7). A population-based study conducted in Italy further demonstrated that patients with diabetes have a two- to three-fold higher risk of mortality from chronic liver diseases, particularly MASLD, compared to non-diabetic individuals (8). Conversely, findings from a 10-year cohort study revealed that histological improvement in MASLD was independently associated with a nearly 70% reduction in the risk of incident T2DM (9). Collectively, these findings underscore the clinical importance of early detection and longitudinal monitoring of MASLD in the management of patients with T2DM.

A variety of imaging modalities are currently available for the screening and diagnosis of MASLD; however, each method presents specific limitations that hinder their widespread application. Ultrasound and computed tomography (CT) are commonly used non-invasive tools for initial MASLD assessment (10). Nevertheless, ultrasound results are susceptible to operator-dependent variability and can be influenced by factors such as abdominal adiposity and disease staging (11). CT, although less operator-dependent, exposes patients to ionizing radiation, has limited sensitivity for detecting early or mild hepatic steatosis, and is associated with relatively high costs, restricting its routine use (12). Magnetic resonance imaging (MRI) and its advanced techniques, including proton density fat fraction (PDFF), chemical shift (including encoded MRI and magnetic resonance spectroscopy), offer high accuracy and reproducibility in quantifying hepatic fat content. However, these methods are time-intensive, expensive, and contraindicated in certain clinical settings (e.g., acute intracranial hemorrhage), thus rendering them unsuitable for large-scale screening (13). The controlled attenuation parameter (CAP), an emerging tool derived from transient elastography, is simple, rapid, and non-invasive. Despite its convenience, CAP accuracy may be compromised by various confounders such as diabetes, obesity, limited intercostal space, hepatic inflammation, and fibrosis (14). Liver biopsy remains the reference standard for definitive diagnosis and histological staging of MASLD. However, its invasive nature introduces procedural risks, including bleeding, infection, and bile leakage, and sampling limitations can lead to false-negative findings (15). Additionally, all of the aforementioned diagnostic modalities must be conducted in clinical settings, further limiting their accessibility for early-stage or asymptomatic individuals. Given these limitations, there is an urgent need to develop a novel screening tool that is non-invasive, reproducible, cost-effective, and easily accessible.

In recent years, clinical prediction models have garnered increasing attention in the medical community due to their high diagnostic accuracy, broad applicability across diverse populations, and operational simplicity. Several models originally developed for NAFLD are now highly relevant for identifying MASLD, as they primarily utilize metabolic parameters. For instance, the Framingham Steatosis Index (FSI) has emerged as a practical tool for identifying hepatic steatosis, achieving areas under the receiver operating characteristic curve (AUCs) of 0.845 and 0.760 in internal and external validation cohorts, respectively (16). Similarly, both the Zhejiang University Index (ZJU) (17) and the Hepatic Steatosis Index (HSI) (18) were developed using data from general adult populations and demonstrated excellent discriminatory power (AUC > 0.80). In addition, the triglyceride-glucose (TyG) index, a low-cost and reliable surrogate marker of insulin resistance (IR), has gained substantial traction in MASLD screening. Numerous large-scale studies leveraging national and regional databases have confirmed the TyG index's predictive utility for MASLD in individuals from the general population (19, 20). However, it is important to note that the majority of existing models were established and validated in general populations and do not account for the unique metabolic characteristics of individuals with T2DM. These models often assume a uniform MASLD risk across all subgroups, which may result in suboptimal risk estimation in diabetic populations. Given the distinct pathophysiological interplay between IR, β-cell dysfunction, and hepatic steatosis in T2DM, there remains a critical need to develop tailored prediction tools that address the specific risk factors and clinical profiles of this high-risk group.

In light of the practical demands of T2DM management and the aforementioned limitations of existing screening models, the present study aimed to develop a novel, individualized, and clinically applicable diagnostic tool for MASLD tailored specifically to the T2DM population. This tool, the Diagnostic Predictive Nomogram (DPN), was constructed using readily available demographic, anthropometric, and laboratory parameters. To ensure its robustness and clinical utility, the model's predictive performance was rigorously evaluated and externally validated, with the goal of facilitating broader applicability across diverse clinical settings.

## 2 Materials and methods

### 2.1 Study population

All data used in this study were obtained from hospitalized patients. Data were retrospectively extracted from the Hospital Information System (HIS) of the First Hospital of Lanzhou University, covering the period from January 2016 to June 2022. Eligible participants met the following inclusion criteria: (1) confirmed diagnosis of T2DM prior to hospital admission, based on the diagnostic criteria of the World Health Organization (WHO) (21); (2) age≥18 years; and (3) availability of complete clinical baseline and abdominal ultrasound imaging data. Exclusion criteria were as follows: (1) diagnosis of T1DM, gestational diabetes mellitus (GDM), or other specific types of diabetes; (2) presence of acute diabetic complications, such as diabetic ketoacidosis or hyperosmolar hyperglycaemic coma; (3) current diagnosis of drug-induced hepatitis, viral hepatitis, or autoimmune hepatitis; (4) missing data on alcohol consumption or documented history of alcohol abuse/excessive drinking; (5) presence of insulinoma, pituitary adenoma, Cushing's syndrome, or secondary pancreatic injury; (6) current use of medications known to affect glucose metabolism, including thyroid hormones, glucocorticoids, or growth hormones; (7) presence of severe infections, stress-related conditions, or cancer cachexia; and (8) prior history of fatty liver disease. This study was approved by the Ethics Committee of the First Hospital of Lanzhou University (Approval No. LDYYLL2021-294). Written informed consent was obtained from all participants. The study was conducted in accordance with the Transparent Reporting of a multivariable prediction model for Individual Prognosis Or Diagnosis (TRIPOD) guidelines (22).

### 2.2 Patient screening process

**Figure 1:**
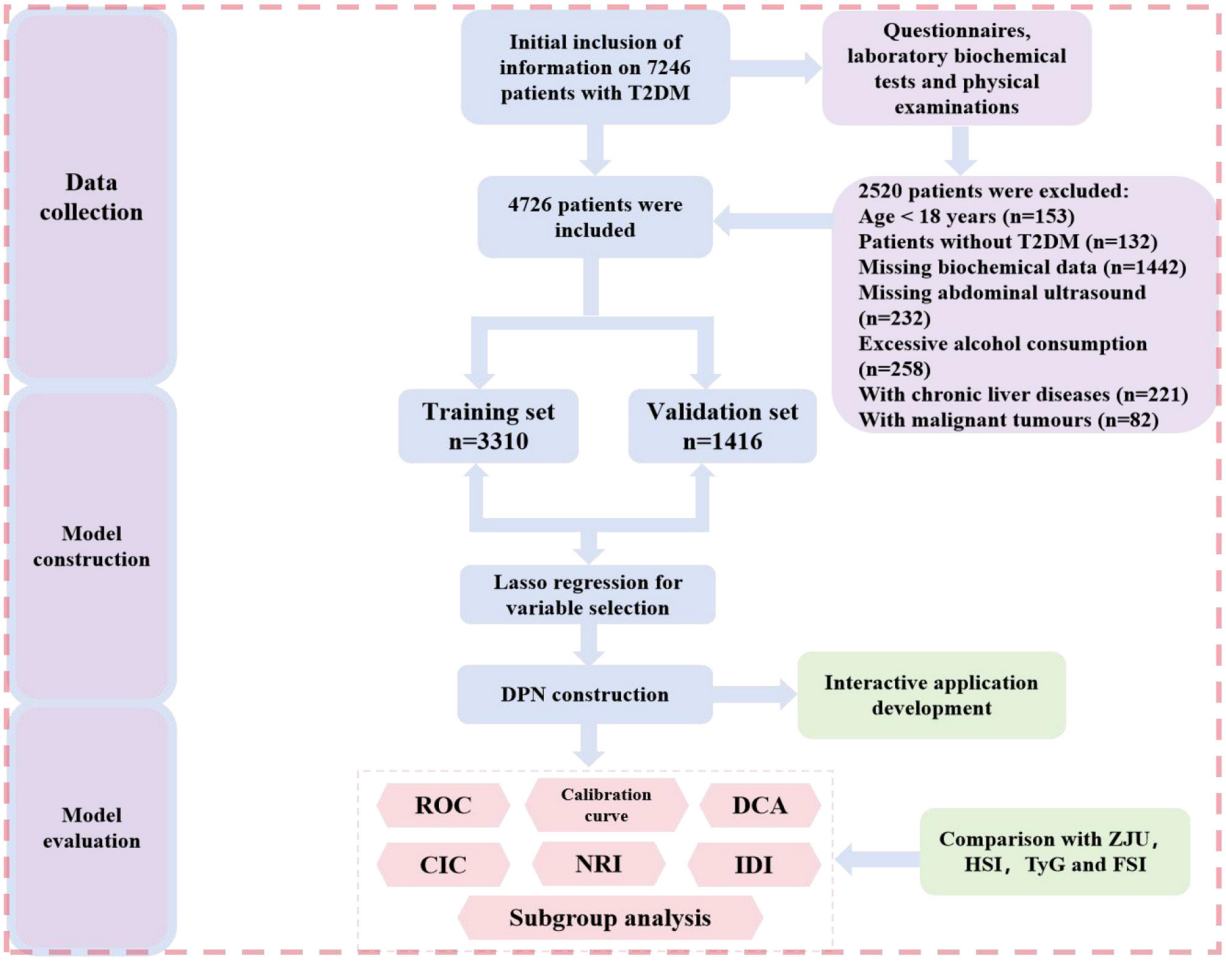
Flowchart of the study design.

A total of 7,246 participants were initially identified. After applying the predefined inclusion and exclusion criteria, the following individuals were excluded: those aged < 18 years (*n* = 153), those with non-T2DM diagnoses (*n* = 132), individuals with missing key biochemical data (*n* = 1,442), those without abdominal ultrasound records (*n* = 232), individuals with a history of excessive alcohol consumption (*n* = 258), those diagnosed with chronic liver diseases (*n* = 221), and patients with malignant tumors (*n* = 82). After exclusions, a final cohort of 4,726 patients with confirmed T2DM was included in the analysis. A detailed flowchart of the participant selection process is presented in Figure 1. All 4,726 hospitalized T2DM patients included in this study were admitted due to diabetes-related complications. The primary indications for hospitalization included diabetic distal symmetrical polyneuropathy (DSPN, 89.9%), diabetic peripheral vascular disease (DPVD, 68.5%), and diabetic nephropathy (DN, 61.2%). Among the common comorbidities, 1,756 patients had MASLD (originally diagnosed as fatty liver/NAFLD). Concurrently, hypertension (51.8%), hyperlipidaemia (25.3%), atherosclerosis (15.2%), and cardiovascular diseases (23.1%) were the major coexisting conditions, collectively constituting the clinical indications associated with hospitalization.

### 2.3 Sample estimation

It is generally recommended that a minimum of 10 outcome events be available for each candidate variable considered in the development of a predictive model (23). In this study, 29 variables were planned for inclusion, necessitating at least 290 outcome events. Given the reported prevalence of MASLD among patients with T2DM ranges from 50% to 75%, the estimated minimum required sample size would range from 387 to 580 individuals. The final cohort of 4,726 patients included in this study meets this requirement, thereby ensuring adequate statistical power for model development.

### 2.4 Data collection

This study collected comprehensive clinical data from hospitalized patients with T2DM, including demographic characteristics, anthropometric parameters, and biochemical indicators. Demographic data encompassed sex, age, duration of diabetes, smoking and alcohol consumption history, as well as medical history related to hypertension and diabetes-associated complications. These variables were obtained through standardized questionnaires administered by trained healthcare professionals. Anthropometric measurements, including height, weight, systolic blood pressure (SBP), and diastolic blood pressure (DBP), were recorded by certified staff using calibrated instruments and standardized protocols. Height and weight were measured using an automatic anthropometer (accuracy: 0.1 cm and 0.1 kg, respectively), and body mass index (BMI) was calculated as weight divided by height squared (kg/m^2^). Blood pressure was measured in the seated position after a 5–10 min rest period; three consecutive readings were taken using an electronic sphygmomanometer, and the average was used for analysis. Biochemical parameters, including comprising fasting blood glucose (FBG), uric acid (UA), alanine aminotransferase (ALT), aspartate aminotransferase (AST), alkaline phosphatase (ALP), γ-glutamyl transpeptidase (GGT), total bilirubin (TBIL), direct bilirubin (DBIL), indirect bilirubin (IBIL), total protein (TP), albumin (ALB), globulin (GLB), total cholesterol (TC), triglycerides (TG), high-density lipoprotein cholesterol (HDL-C), and low-density lipoprotein cholesterol (LDL-C), were measured using venous blood samples collected after an overnight fast of at least 8 h. All samples were analyzed in a central laboratory using a fully automated biochemical analyzer (LABOSPECT, Japan), with all procedures performed under standardized conditions to ensure consistency and accuracy.

### 2.5 Research outcomes

The primary outcome of this study was the first-time diagnosis of MASLD during the index hospitalization, with no prior history of hepatic steatosis or related liver conditions. MASLD was diagnosed by two experienced ultrasound physicians using a high-resolution ultrasound imaging system, based on standardized diagnostic criteria. Characteristic imaging features included: (1) an intact liver capsule with a significantly enlarged, rounded liver contour; (2) dense echogenic foci diffusely distributed within the hepatic parenchyma, with attenuation of echo intensity from superficial to deep regions, and a foggy appearance of the diaphragm and liver base due to impaired sound transmission; (3) reduced visibility of intrahepatic tubular structures, diminished vascular echoes, blurred vascular branches, and a decreased liver-to-kidney parenchymal echogenicity ratio; and (4) in cases of diffuse hepatic steatosis, a homogeneous distribution of steatosis throughout the liver with uniformly increased echogenicity and no evidence of focal heterogeneity. The diagnosis of MASLD was assigned to patients who exhibited these imaging features and had pre-existing T2DM—a cardinal criterion for metabolic dysfunction—after the exclusion of other identifiable causes of fatty liver, including alcohol abuse, viral hepatitis, drug-induced liver disease, or total parenteral nutrition (24).

### 2.6 Complications and concomitant conditions

Hypertension was defined as a SBP ≥140 mmHg and/or DBP ≥90 mmHg on three separate occasions in the absence of antihypertensive therapy. Individuals with a prior history of hypertension who maintained blood pressure below 140/90 mmHg while on antihypertensive medications were still classified as hypertensive (25). DN was diagnosed in patients with type 2 diabetes mellitus after excluding other causes of chronic kidney disease, based on an estimated glomerular filtration rate (eGFR) < 60 mL/min/1.73 m^2^ or a urinary albumin-to-creatinine ratio >30 mg/g persisting for more than 3 months (26). DSPN was defined as a neuropathy occurring after the onset of diabetes and diagnosed when patients exhibited at least two of the following clinical features: impaired temperature or vibration sensation, slowed nerve conduction velocity, reduced or absent distal sensation in the feet, or diminished ankle reflexes (27). DPVD was defined as peripheral arterial atherosclerosis confirmed by ultrasound imaging in the context of a known diagnosis of diabetes mellitus (28).

### 2.7 Existing prediction models for fatty liver disease

In order to evaluate the performance of the model we developed, this study also comprehensively compared the performance of DPN with four previously reported prediction models for fatty liver disease. These four models are:

(1) ZJU = BMI (kg/m^2^) + FPG (mmol/L) + TG (mmol/L) + 3 × [ALT(U/L)/AST(U/L)] (+ 2, if for women);
(2) HSI = 8 × [ALT(U/L)/AST(U/L)] + BMI (kg/m^2^)(+ 2, if T2DM; + 2, if for women);
(3) TyG = Ln [TG (mg/dL) × FPG (mg/dL)/2];
(4) FSI = −7.981 + 0.011 × Age (years) – 0.146 × Sex (female = 1, male = 0) + 0.173 × BMI (kg/m^2^) + 0.007 × TG (mg/dL)+ 0.593 × Hypertension (yes = 1, no = 0) + 0.789 × T2DM (yes = 1, no = 0) + 11 × [ALT(U/L)/AST(U/L)] > 1.33 (yes = 1, no = 0).

### 2.8 Model establishment

The study cohort comprising 4,726 patients was randomly divided into a training set (*n* = 3,310) and a validation set (*n* = 1,416) in a 7:3 ratio using the “Caret” package in R. The DPN was constructed based on the training set. To preliminarily explore associations between candidate variables and MASLD, univariate logistic regression analyses were performed. To further reduce dimensionality, eliminate irrelevant or multicollinear variables, and prevent model overfitting, least absolute shrinkage and selection operator (LASSO) regression was applied using the “glmnet” package with 10-fold cross-validation. Variables with non-zero coefficients were retained as potential predictors. The regularization parameter (lambda.lse), corresponding to the minimum mean squared error and optimal model performance, was selected for subsequent analysis. Following LASSO selection, variance inflation factors (VIFs) were calculated to assess multicollinearity among the remaining variables, and restricted cubic spline (RCS) analyses were conducted to explore potential non-linear relationships between continuous predictors and MASLD risk. Subsequently, multivariate logistic regression with stepwise variable selection based on the Akaike Information Criterion (AIC) was performed using the “MASS” package to develop the final prediction model. To enhance clinical utility and accessibility, an interactive web-based calculator was developed using the “Shiny” package in R. Additionally, a probability reference table based on the DPN was provided in the Supplementary material for practical application in healthcare settings.

### 2.9 Evaluation of model performance

The performance of the DPN was evaluated from three perspectives: discrimination, calibration, and clinical utility. First, the discriminative ability of the model was assessed by plotting receiver operating characteristic (ROC) curves using the “pROC” package in R. The AUC was calculated to quantify the ability of DPN, as well as existing models including the ZJU, HSI, TyG, and FSI, to distinguish MASLD in patients with T2DM. The Delong test was applied to compare AUCs between models, with statistical significance defined as *P* < 0.05. To further evaluate incremental predictive value, the net reclassification index (NRI) and integrated discrimination improvement (IDI) were computed. Additional diagnostic performance metrics derived from ROC analysis, including sensitivity (SEN), specificity (SPE), positive predictive value (PPV), negative predictive value (NPV), positive likelihood ratio (PLR), negative likelihood ratio (NLR), diagnostic odds ratio (DOR), Youden's index, and optimal cutoff value, were also calculated. Second, model calibration was assessed using calibration plots generated with the “calibrate” package to compare predicted probabilities with observed outcomes. A Brier score < 0.25 and a non-significant Hosmer–Lemeshow test (*P* > 0.05) were considered indicative of good calibration. Finally, the clinical utility of the DPN was evaluated using decision curve analysis (DCA) and clinical impact curves (CIC) to compare the net clinical benefit across various threshold probabilities against those of the ZJU, HSI, TyG, and FSI models. The DCA and CIC plots were generated using the “dca.R” function in R.

### 2.10 Model validation

To evaluate the generalizability and robustness of the diagnostic predictive nomogram (DPN), both internal and external validation were performed. Internal validation was conducted using the previously defined validation subset of the study cohort (*n* = 1,416). For external validation, an independent dataset was extracted from the National Health and Nutrition Examination Survey (NHANES) database, covering the period from 2017 to March 2020, comprising 471 eligible individuals. Details of the validation process are presented in Supplementary Figure S1. NHANES is a nationally representative, cross-sectional survey conducted by the National Center for Health Statistics (NCHS) to assess the health and nutritional status of the U.S. population. The survey protocol was approved by the NCHS Research Ethics Review Board, and all participants provided written informed consent. Additional information regarding NHANES is available on the official website: https://www.cdc.gov/nchs/nhanes/index.html.

### 2.11 Statistical analysis

All statistical analyses and graphing were performed using SPSS 27.0 (IBM Corp., Armonk, NY, USA) and R software 4.3.1 (R Foundation for Statistical Computing, Vienna, Austria). Among the 29 predictive variables included, the proportion of missing values was less than 30% for all variables. Therefore, we imputed missing values using the k-nearest neighbor algorithm (KNN). For the description of baseline data, when continuous variables were normally distributed, they were expressed as mean ± standard deviation, and comparisons between groups were performed using *t*-tests. When the measurement data were not normally distributed, the median and interquartile range [M (P25-P75)] were used, and the Mann-Whitney U test was used for comparisons between the two groups. The count data were expressed as frequencies and percentages, and the chi-square test was used for comparisons between the two groups. A *P*-*value* < 0.05 was considered statistically significant.

## 3 Results

### 3.1 Basic characteristics of the research subjects

This study initially included 7,246 patients, with 4,726 meeting the inclusion criteria. The inclusion process is shown in Figure 1. Among the 4,726 patients with T2DM, the average age was 59.43 years, with 61.3% being male (*n* = 2,901). The enrolled population was divided into the MASLD group (*n* = 1,756) and the non-MASLD group (*n* = 2,970) based on whether MASLD was first diagnosed during hospitalization. Table 1 describes the basic characteristics and metabolic parameters of the two groups of patients. Compared with the non-MASLD group, the MASLD group had higher body weight and BMI, younger age, shorter disease duration, and lower proportions of males, DSPN, and DPVD (*P* < 0.05). Moreover, the MASLD group had higher levels of DBP, FBG, TC, TG, LDL-C, UA, ALT, AST, ALP, TP, ALB, TBIL, DBIL, and IBIL, and lower levels of HDL-C and GLB (*P* < 0.05). Notably, the four MASLD-related predictive parameters (ZJU, HSI, TyG, and FSI) were higher in the MASLD group than in the non-MASLD group (*P* < 0.001). For the development and validation of the predictive model, the patients were randomly divided into the training set (*n* = 3,310) and the validation set (*n* = 1,416) in a 7:3 ratio. The average age of patients in the training set was 59.65 years, with 2,039 patients (61.6%) being male, and the prevalence of MASLD was 37.07% (*n* = 1,227). Patients in the validation set were similar to those in the training set in most clinical characteristics, with no significant differences between the two groups (*P3* > 0.05, see Supplementary Table S1).

**Table 1:** Baseline characteristics of participants with T2DM.

**Variables**	**Overall (*n* = 4,726)**	**Non-MASLD (*n* = 2,970)**	**MASLD (*n* = 1,756)**	***P*-*value***
Age (years)	59.43 ± 11.86	61.23 ± 11.31	56.39 ± 12.14	< 0.001
Male *n*, (%)	2,901 (61.3)	1,856 (62.4)	1,045 (59.5)	0.042
Height (cm)	168 (160–173)	168 (160–173)	168 (161–173)	0.071
Weight (kg)	68.73 ± 11.20	66.46 ± 10.47	72.57 ± 11.36	< 0.001
BMI (kg/m2)	24.53 ± 3.11	23.79 ± 2.91	25.79 ± 3.03	< 0.001
Course (years)	8 (3–13)	10 (4–15)	5 (1–10)	< 0.001
Smoking *n*, (%)	755 (15.9)	455 (15.3)	300 (17.0)	0.110
Hypertemsion *n*, (%)	2,448 (51.7)	1,561 (52.5)	887 (50.5)	0.174
DN *n*, (%)	2,894 (61.2)	1,811 (61.0)	1,083 (61.7)	0.634
DSPN *n*, (%)	4,248 (89.8)	2,697 (90.8)	1,551 (88.3)	0.006
DPVD *n*, (%)	3,238 (68.5)	2,122 (71.4)	1,116 (63.6)	< 0.001
SBP (mmHg)	137.19 ± 21.04	137.04 ± 21.86	137.45 ± 19.57	0.514
DBP (mmHg)	82.53 ± 11.52	81.33 ± 11.61	84.57 ± 11.09	< 0.001
FBG (mmol/L)	9.96 (7.45–13.94)	9.70 (7.29–13.81)	10.34 (7.84–14.20)	< 0.001
TC (mmol/L)	4.21 (3.54–4.94)	4.13 (3.47–4.84)	4.34 (3.69–5.09)	< 0.001
TG (mmol/L)	1.66 (1.16–2.43)	1.47 (1.05–2.12)	2.00 (1.45–2.98)	< 0.001
HDL-C (mmol/L)	1.13 (0.95–1.32)	1.16 (0.98–1.37)	1.07 (0.91–1.26)	< 0.001
LDL-C (mmol/L)	2.69 (2.08–3.33)	2.66 (2.03–3.28)	2.74 (2.14–3.42)	< 0.001
UA (umol/L)	309 (256–366)	300 (251–356)	324 (267–379)	< 0.001
ALT (U/L)	21 (15–31)	19 (14–27)	25 (18–38)	< 0.001
AST (U/L)	20 (16–25)	19 (16–24)	21 (17–28)	< 0.001
GGT (U/L)	28 (20–43)	25 (18–37)	34 (24–52)	< 0.001
ALP (U/L)	83 (69–102)	82 (68–101)	85 (69–104)	0.007
TP (g/L)	74.9 (71.3–78.5)	74.5 (70.7–77.9)	75.7 (72.3–79.1)	< 0.001
ALB (g/L)	45.1 (42.4–47.5)	44.5 (41.7–46.9)	46.0 (43.5–48.2)	< 0.001
GLB (g/L)	29.7 (26.5–33.2)	29.8 (26.7–33.3)	29.5 (26.3–33.1)	0.031
TBIL (umol/L)	12.8 (9.7–17.1)	12.5 (9.4–16.6)	13.5 (10.3–17.9)	< 0.001
DBIL (umol/L)	4.8 (3.6–6.4)	4.7 (3.5–6.3)	5.0 (3.8–6.6)	< 0.001
IBIL (umol/L)	7.9 (5.6–11.1)	7.6 (5.4–10.7)	8.4 (6.1–11.9)	< 0.001
ZJU	40.99 (37.05–45.70)	39.70 (35.81–44.18)	43.35 (39.42–47.66)	< 0.001
HSI	36.00 (32.93–39.36)	34.71 (31.95–37.71)	38.26 (35.38–41.54)	< 0.001
TyG	9.51 (9.01–10.07)	9.38 (8.86–9.93)	9.73 (9.27–10.25)	< 0.001
FSI	−0.64 (−1.38 to 0.22)	−0.95 (−1.58 to −0.18)	−0.11 (−0.81 to 0.78)	< 0.001

### 3.2 Screening of important variables in the training set

**Figure 2:**
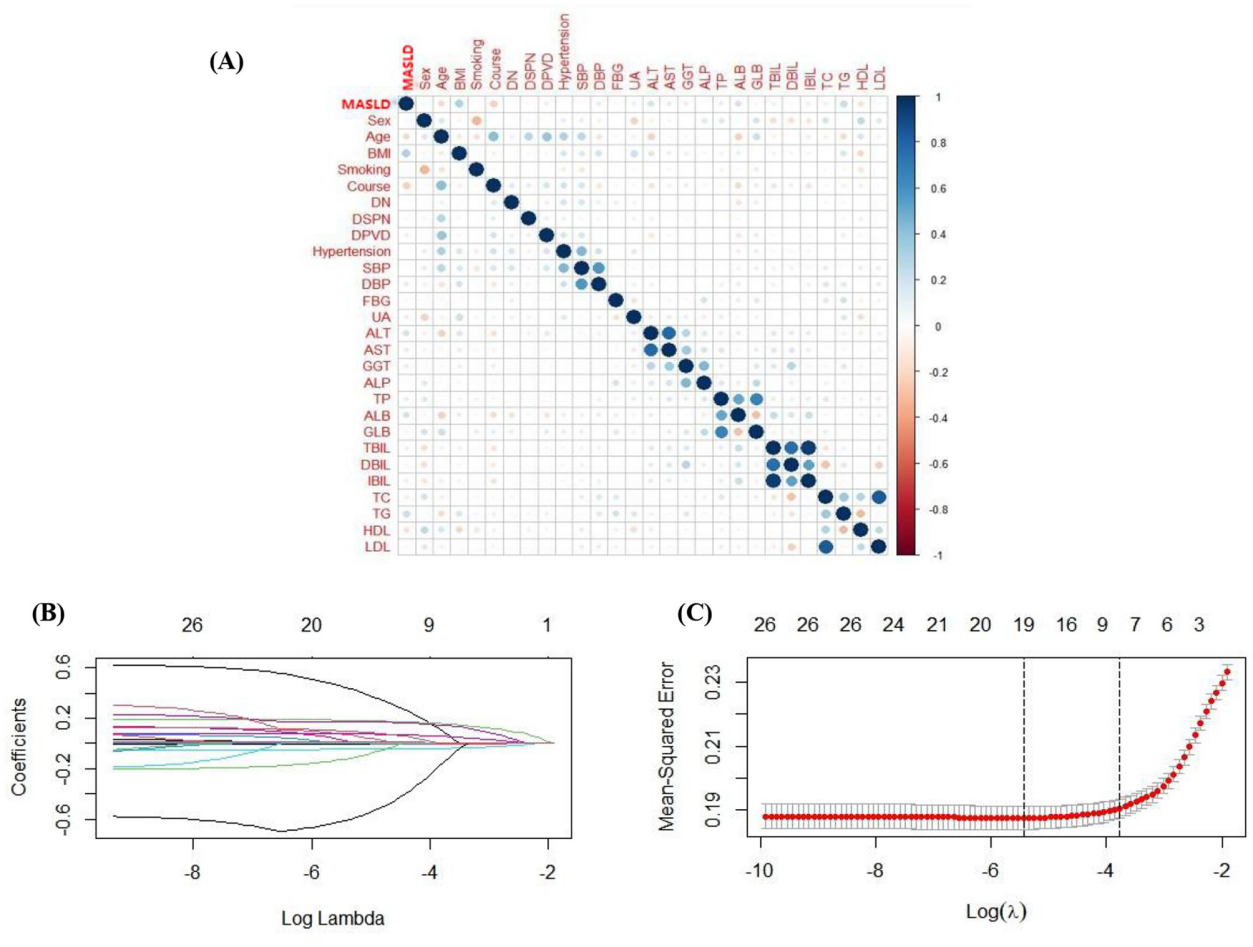
Correlation heat map and variable selection in the training set. **(A)** The correlation heat map is used to determine the relationship between variables. **(B)** Select the optimal parameter λ in LASSO regression, with the x-axis representing logλ and the y-axis representing the coefficient. **(C)** Relationship with the number of variables, with the lower end of the x-axis representing logλ, the upper end of the x-axis representing the number of variables, and the y-axis representing the mean square error.

A correlation heatmap was used to preliminarily assess the association between the aforementioned 27 variables (excluding height and weight, which are closely related to BMI) related to demographic factors, laboratory factors, anthropometric measurements, lifestyle, comorbidities, and MASLD (Figure 2A). We further used univariate logistic regression to quantify the association between the aforementioned variables and MASLD in patients with T2DM, with 22 variables showing a significant association with MASLD (Supplementary Table S2, *P* < 0.05). To ultimately develop a model with the fewest variables, no overfitting, and optimal performance, we used the LASSO algorithm to perform dimensionality reduction and variable selection on the aforementioned 27 variables, selecting an appropriate penalty coefficient λ via 10-fold cross-validation. As the penalty coefficient λ increased, the coefficients of some of the included variables were gradually compressed to 0 (Figure 2B). At λ = 0.0232 (1 standard error of λ), the number of selected variables was minimal, and the mean squared error was small (Figure 2C). At this point, 8 variables with non-zero coefficients were obtained, namely gender, age, BMI, ALT, ALB, disease duration, TG, and HDL (Supplementary Table S2). Their VIF values ranged from a maximum of 1.272 to a minimum of 1.029, with all VIF values < 5, indicating no multicollinearity among them.

### 3.3 Establishment of DPN in the training set

**Figure 3:**
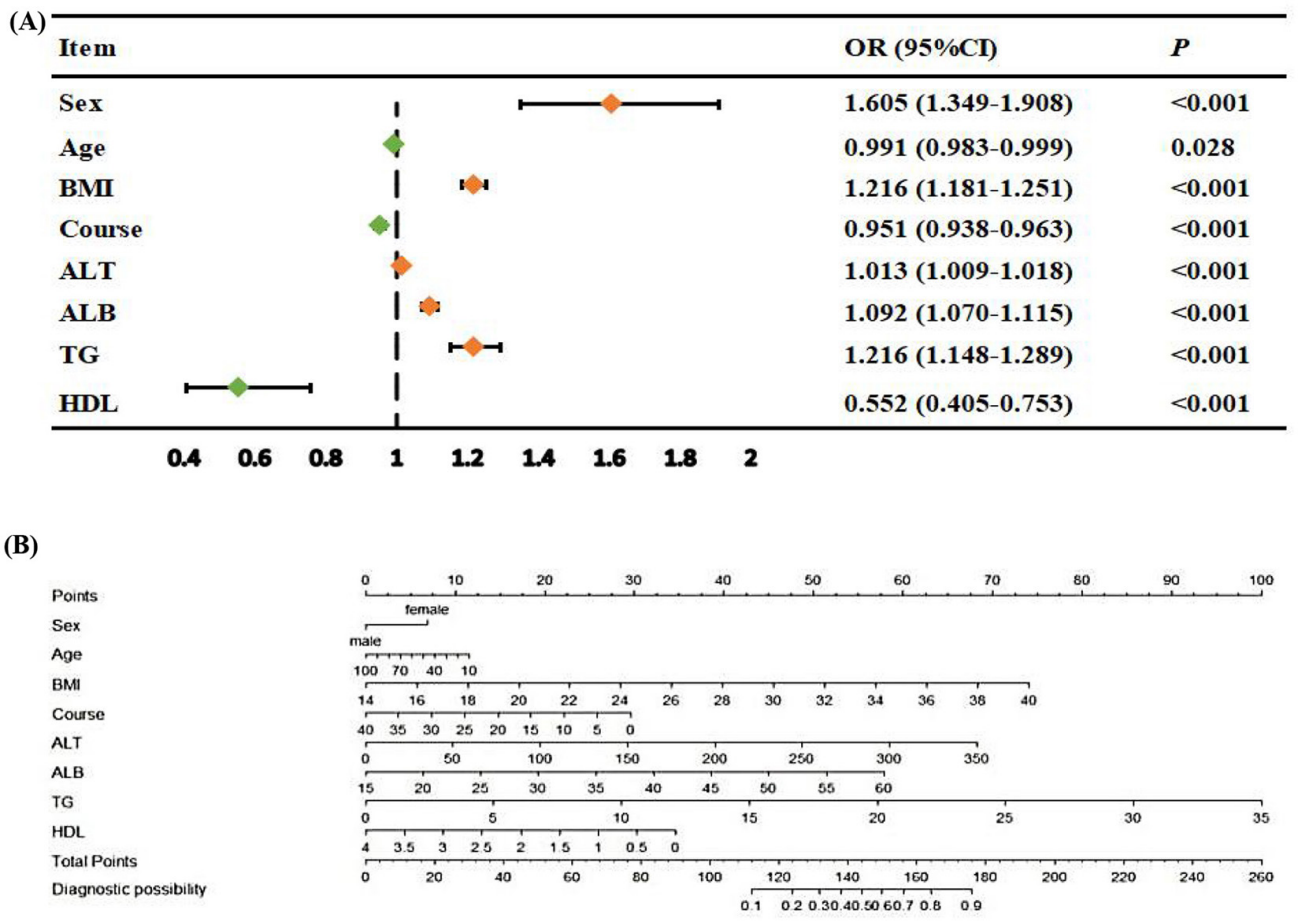
Forest plot and diagnostic prediction nomogram. **(A)** Forest plot visualization of the results of stepwise AIC-selected multiple logistic regression. **(B)** DPN shows the predicted probability of MASLD, ranging from 0 to 200. A vertical line is drawn for each predictor, and the corresponding score is recorded. The scores for each predictor are added together to obtain the total score. The total score is the predicted probability of MASLD.

After LASSO regression, the aforementioned eight predictive factors were selected as the optimal variables. These eight variables were further incorporated into a stepwise AIC-based multivariate logistic regression analysis, which revealed that gender, age, BMI, ALT, ALB, disease duration, TG, and HDL were all statistically significant (*P* < 0.05, Figure 3A and Supplementary Table S3). RCS analysis further confirmed that the continuous variables among the aforementioned predictive factors (age, BMI, ALT, ALB, disease duration, TG, and HDL) exhibited either a nonlinear relationship (*P*_overall_ < 0.05, *P*_non − linear_ < 0.05) or a linear relationship (*P*_overall_ < 0.05, *P*_non − linear_ > 0.05) with MASLD risk, as shown in Supplementary Figure S2. These variables were used to construct the DPN and presented in the form of nomogram that quantifies MASLD risk (Figure 3B). Moreover, for the convenience of clinical medical staff, we further developed an interactive application based on the R package Shiny (a MASLD probability scale based on this program is also included in the Supplementary material). The uniform resource locator (URL) for this program is https://wangyazhi.shinyapps.io/DynNomapp/. The model summary is shown in Supplementary Figure S3.

### 3.4 Performance evaluation of DPN

ZJU, HSI, TyG, and FSI are several previously reported predictive models that are widely used in the assessment of fatty liver disease, including MASLD. To comprehensively evaluate the performance, applicability, and stability of DPN, this study calculated and compared multiple performance metrics, including ROC curves, SEN, and SPE across the training set, validation set (internal validation set), and test set (external validation set from the NHANES database). We also conducted calibration and clinical utility assessments, as well as subgroup analyses.

### 3.5 Comparison of performance parameters between DPN and other existing models

**Figure 4:**
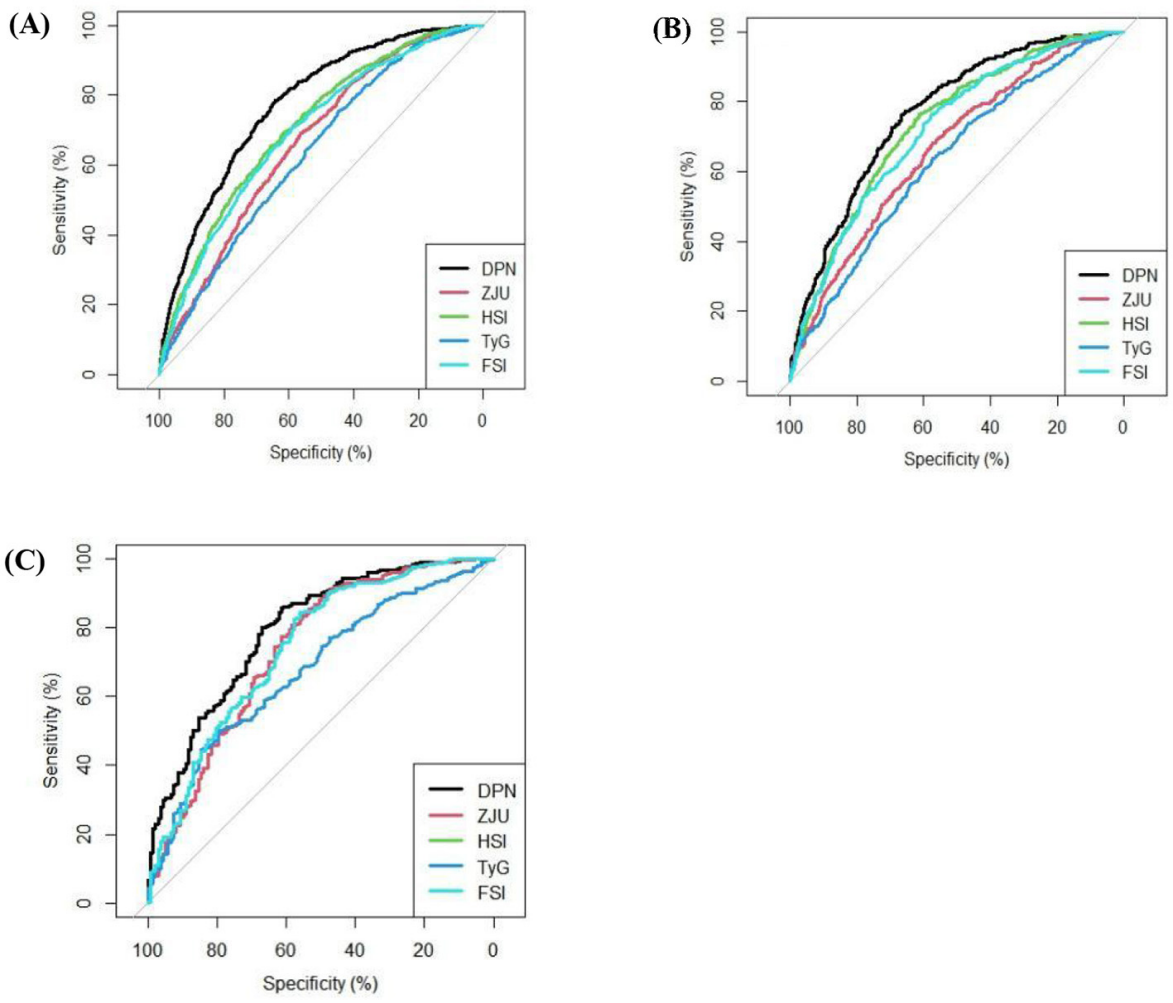
ROC curves of DPN and other models on the training set **(A)**, validation set **(B)**, and NHANES dataset **(C)**.

Compared with ZJU, HSI, TyG, and FSI, DPN had the highest AUC values for predicting MASLD risk in the training set (0.775, 95% CI: 0.759–0.791), validation set (0.767, 95% CI: 0.742–0.791) and test set (0.794, 95% CI: 0.749–0.839), and these AUC values were statistically significantly different from those of the aforementioned four models (*P* < 0.05), as shown in Tables 2–4 and Figure 4. This indicated that DPN has the best discriminatory ability for MASLD and non-MASLD. Moreover, DPN demonstrated excellent SEN and SPE for predicting MASLD risk in the training set (SEN 0.785, 95% CI: 0.761–0.807; SPE 0.640, 95% CI: 0.619–0.661), the validation set (SEN 0.760, 95% CI: 0.721–0.795; SPE 0.666, 95% CI: 0.634–0.697), and test set (SEN 0.799, 95% CI: 0.752–0.840; SPE 0.672, 95% CI: 0.585–0.748). Compared to other models, Youden's index and DOR were the highest. Tables 2–4 described the specific details of other performance parameters (including PPV, NPV, PLR, NLR, and cutoff values). Moreover, alternative rule-in/rule-out cut-offs are provided in the Supplementary Table S4.

**Table 2:** Comparison of the performance of DPN and other existing models in predicting MASLD in the training set.

**Models**	**AUC (95% CI)**	***P*1 values**	**SEN (95% CI)**	**SPE (95% CI)**	**PPV (95% CI)**	**NPV (95% CI)**	**PLR (95% CI)**	**NLR (95% CI)**	**DOR**	**Youden**	**Cut off values**	**NRI (95% CI)**	***P*2 values**	**IDI (95% CI)**	***P*3 values**
DPN	0.775 (0.759–0.791)	–	0.785 (0.761–0.807)	0.640 (0.619–0.661)	0.562 (0.538–0.586)	0.835 (0.815–0.852)	2.180 (2.044–2.325)	0.336 (0.302–0.374)	6.488	0.425	−0.761	Ref	–	Ref	–
ZJU	0.665 (0.647–0.684)	< 0.001	0.689 (0.662–0.714)	0.564 (0.542–0.585)	0.482 (0.458–0.506)	0.755 (0.732–0.776)	1.580 (1.485–1.680)	0.552 (0.507–0.600)	2.862	0.253	40.785	0.213 (0.177–0.248)	< 0.001	0.132 (0.120–0.145)	< 0.001
HSI	0.711 (0.693–0.729)	< 0.001	0.652 (0.624–0.679)	0.656 (0.635–0.676)	0.527 (0.502–0.553)	0.762 (0.741–0.781)	1.894 (1.763–2.036)	0.762 (0.741–0.781)	2.486	0.308	36.525	0.102 (0.070–0.134)	< 0.001	0.083 (0.072–0.093)	< 0.001
TyG	0.635 (0.616–0.654)	< 0.001	0.757 (0.732–0.781)	0.446 (0.425–0.468)	0.446 (0.425–0.468)	0.757 (0.732–0.781)	1.367 (1.300–1.437)	0.545 (0.493–0.602)	2.508	0.203	9.268	0.243 (0.206–0.278)	< 0.001	0.154 (0.141–0.168)	< 0.001
FSI	0.696 (0.677–0.714)	< 0.001	0.641 (0.614–0.668)	0.656 (0.635–0.676)	0.523 (0.498–0.549)	0.756 (0.736–0.776)	1.863 (1.733–2.004)	0.547 (0.507–0.590)	3.406	0.297	−0.496	0.150 (0.118–0.182)	< 0.001	0.107 (0.096–0.118)	< 0.001

DPN, diagnostic predictive nomogram; ZJU, the ZJU index; HSI, hepatic steatosis index; TyG, triglyceride-glucose index; FSI, Framingham steatosis index; AUC, area under the curve; SEN, sensitivity; SPE, specificity; PPV, positive predictive value; NPV, negative predictive value; PLR, positive likelihood ratio; NLR, negative likelihood ratio; DOR, diagnostic odds ratio; NRI, net reclassification index; IDI, integrated discrimination improvement.

**Table 3:** Comparison of the performance of DPN and other existing models in predicting MASLD in the validation set.

**Models**	**AUC (95% CI)**	***P*1 values**	**SEN (95% CI)**	**SPE (95% CI)**	**PPV (95% CI)**	**NPV (95% CI)**	**PLR (95% CI)**	**NLR (95% CI)**	**DOR**	**Youden**	**Cut off values**	**NRI (95% CI)**	***P*2 values**	**IDI (95% CI)**	***P*3 values**
DPN	0.767 (0.742–0.791)	–	0.760 (0.721–0.795)	0.666 (0.634–0.697)	0.576 (0.538–0.613)	0.823 (0.793–0.850)	2.277 (2.051–2.528)	0.360 (0.309–0.419)	6.325	0.426	−0.711	Ref	–	Ref	–
ZJU	0.667 (0.638–0.695)	< 0.001	0.681 (0.639–0.720)	0.573 (0.539–0.605)	0.487 (0.451–0.524)	0.513 (0.476–0.549)	1.593 (1.447–1.753)	0.558 (0.492–0.633)	2.855	0.254	40.130	0.144 (0.091–0.198)	< 0.001	0.110 (0.092–0.128)	< 0.001
HSI	0.732 (0.706–0.758)	0.002	0.764 (0.725–0.799)	0.613 (0.580–0.645)	0.541 (0.504–0.577)	0.813 (0.781–0.842)	1.975 (1.795–2.173)	0.385 (0.330–0.450)	5.130	0.377	35.605	0.052 (0.004–0.100)	0.035	0.049 (0.033–0.065)	< 0.001
TyG	0.634 (0.605–0.664)	< 0.001	0.652 (0.610–0.692)	0.559 (0.526–0.592)	0.469 (0.432–0.506)	0.729 (0.694–0.762)	1.479 (1.343–1.630)	0.622 (0.553–0.700)	2.378	0.211	9.460	0.197 (0.143–0.251)	< 0.001	0.136 (0.116–0.156)	< 0.001
FSI	0.719 (0.692–0.746)	< 0.001	0.764 (0.725–0.799)	0.576 (0.543–0.609)	0.518 (0.482–0.553)	0.803 (0.770–0.833)	1.802 (1.646–1.972)	0.410 (0.351–0.479)	4.395	0.340	−0.812	0.110 (0.064–0.157)	< 0.001	0.075 (0.059–0.091)	< 0.001

DPN, diagnostic predictive nomogram; ZJU, the ZJU index; HSI, hepatic steatosis index; TyG, triglyceride-glucose index; FSI, Framingham steatosis index; AUC, area under the curve; SEN, sensitivity; SPE, specificity; PPV, positive predictive value; NPV, negative predictive value; PLR, positive likelihood ratio; NLR, negative likelihood ratio; DOR, diagnostic odds ratio; NRI, net reclassification index; IDI, integrated discrimination improvement.

**Table 4:** Comparison of the performance of DPN and other existing models in predicting MASLD in the NHANES dataset.

**Models**	**AUC (95% CI)**	***P*1 values**	**SEN (95% CI)**	**SPE (95% CI)**	**PPV (95% CI)**	**NPV (95% CI)**	**PLR (95% CI)**	**NLR (95% CI)**	**DOR**	**Youden**	**Cut off values**	**NRI (95% CI)**	***P*2 values**	**IDI (95% CI)**	***P*3 values**
DPN	0.794 (0.749–0.839)	–	0.799 (0.752-0.840)	0.672 (0.585–0.748)	0.856 (0.811–0.892)	0.579 (0.498–0.656)	2.434 (1.904–3.111)	0.299 (0.239–0.373)	8.140	0.471	−0.890	Ref	–	Ref	–
ZJU	0.737 (0.684–0.790)	0.013	0.808 (0.761–0.848)	0.584 (0.497–0.667)	0.826 (0.779–0.864)	0.556 (0.471–0.638)	1.943 (1.583–2.385)	0.328 (0.261–0.413)	5.924	0.392	41.980	0.126 (0.035–0.218)	0.007	0.091 (0.058–0.124)	< 0.001
HSI	0.742 (0.691–0.793)	0.013	0.844 (0.800–0.881)	0.563 (0.475–0.646)	0.825 (0.779–0.863)	0.597 (0.507–0.681)	1.928 (1.586–2.343)	0.277 (0.213–0.360)	6.960	0.407	38.245	0.147 (0.04–0.247)	0.004	0.084 (0.052–0.116)	< 0.001
TyG	0.677 (0.625–0.729)	< 0.001	0.497 (0.442–0.552)	0.796 (0.717–0.858)	0.856 (0.796–0.900)	0.394 (0.336–0.454)	2.432 (1.718–3.442)	0.632 (0.566–0.706)	3.848	0.293	9.295	0.326 (0.233–0.420)	< 0.001	0.159 (0.121–0.196)	< 0.001
FSI	0.768 (0.720–0.816)	0.117	0.740 (0.688–0.785)	0.701 (0.616–0.774)	0.858 (0.811–0.895)	0.525 (0.450–0.598)	2.471 (1.898–3.218)	0.372 (0.308–0.448)	6.642	0.441	0.205	0.058 (−0.029 to 0.145)	0.188	0.042 (0.016–0.068)	0.002

DPN, diagnostic predictive nomogram; ZJU, the ZJU index; HSI, hepatic steatosis index; TyG, triglyceride-glucose index; FSI, Framingham steatosis index; AUC, area under the curve; SEN, sensitivity; SPE, specificity; PPV, positive predictive value; NPV, negative predictive value; PLR, positive likelihood ratio; NLR, negative likelihood ratio; DOR, diagnostic odds ratio; NRI, net reclassification index; IDI, integrated discrimination improvement.

### 3.6 The incremental value of DPN in identifying MASLD in patients with T2DM

Since the AUC increment is not intuitive or sensitive, this study calculated the NRI and IDI to further assess the predictive capability of DPN compared to other models. In the training set, validation set, and test set, the NRI and IDI of DPN were significantly higher than those of ZJU, HSI, TyG, and FSI (*P* < 0.05), as shown in Tables 2–4. This indicated that DPN has a superior ability to identify the risk of MASLD in the T2DM population compared to existing models.

### 3.7 Assessment of the calibration and clinical utility of DPN

**Figure 5:**
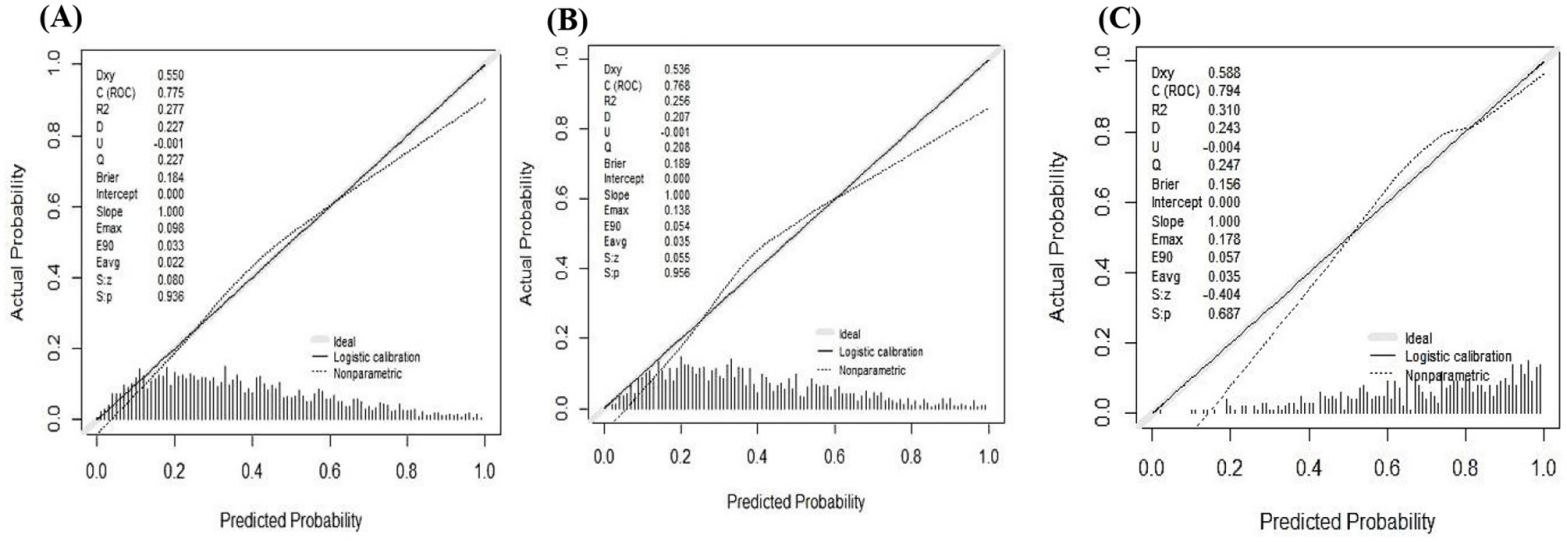
Calibration assessment of DPN in the training set **(A)**, validation set **(B)**, and NHANES set **(C)**. The x-axis represents the predicted probability of MASLD by DPN, and the y-axis represents the actual probability.

**Figure 6:**
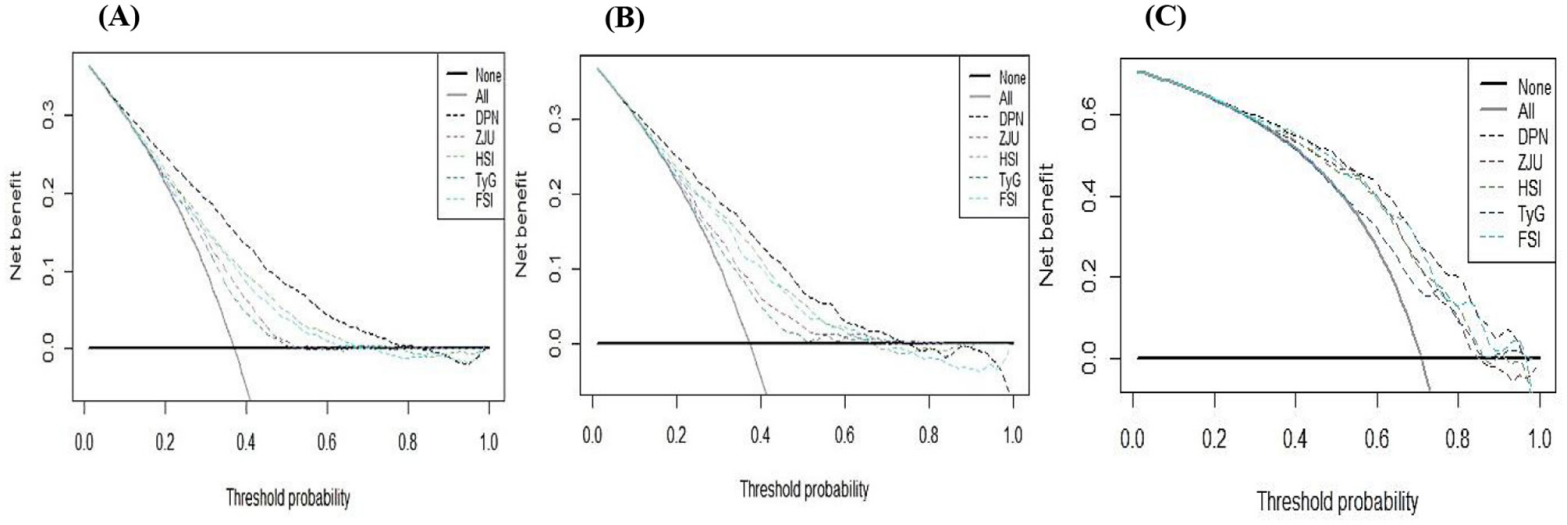
Clinical utility assessment of DPN and other existing models through decision curve analysis in the training set **(A)**, validation set **(B)**, and NHANES set **(C)**. The x-axis represents the threshold probability, and the y-axis represents the net benefit (calculated by subtracting the relative harm from the benefit).

**Figure 7:**
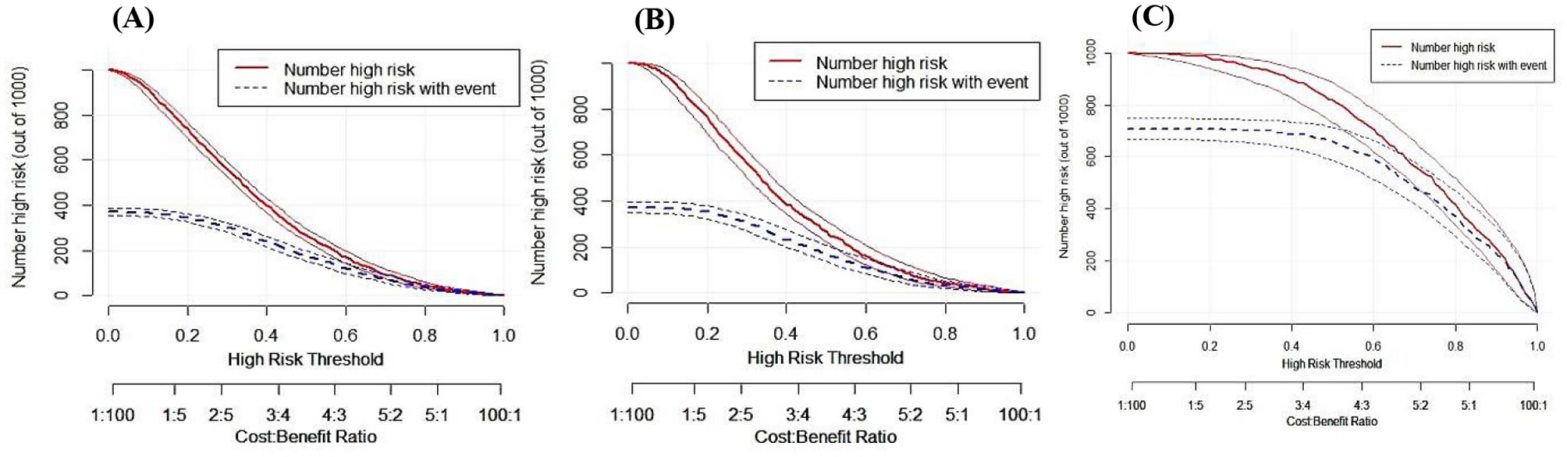
Clinical utility assessment of DPN in the training set **(A)**, validation set **(B)**, and NHANES set **(C)** using clinical impact curves. The x-axis represents the high-risk threshold, and the y-axis represents the number of high-risk individuals.

When the calibration curve closely follows the diagonal line, it indicates that the model's predicted probability is more consistent with the actual probability of disease occurrence, resulting in higher prediction accuracy (*P* > 0.05, Brier score < 0.25). The calibration curves indicated that DPN demonstrated good calibration in the training set (*P* = 0.936, Brier score = 0.184), validation set (*P* = 0.956, Brier score = 0.189), and test set (*P* = 0.687, Brier score = 0.156), as shown in Figure 5. The DCA (Figure 6) and CIC curves (Figure 7) demonstrated the clinical utility of DPN. DCA results indicated that when DPN was within the majority of threshold ranges in the training set (5%−78%), validation set (5%−75%), and test set (11%−98%), its net benefit consistently exceeded that of other predictive models. CIC results suggested that when the threshold probability was ≥60%, the predicted high-risk MASLD population in the training set, validation set, and test set of the T2DM population highly aligned with the actual MASLD incidence population. These findings confirmed the high clinical utility of DPN.

### 3.8 Subgroup analysis

Subgroup analyses confirmed that the DPN exhibited robust discriminatory performance, applicability, and stability across various clinically relevant subgroups, including sex (male and female), age ( ≤ 60 years and >60 years), duration of diabetes ( ≤ 20 years and >20 years), and hypertension status (with and without hypertension) (Supplementary Tables S5–S12; Supplementary Figures S4–S7). In all subgroups, the DPN consistently demonstrated higher AUC values compared with existing models, with most comparisons reaching statistical significance (*P* < 0.05), as shown in Supplementary Figure S4. Furthermore, calibration plots, DCA, and CIC across subgroups reaffirmed that the DPN maintained good calibration and clinical utility irrespective of subgroup stratification (Supplementary Figures S5–S7).

## 4 Discussion

MASLD (formerly known as NAFLD) has become one of the most prevalent chronic liver diseases globally, with its historical prevalence estimated at approximately 25% in the general adult population (29). Among individuals with T2DM, the prevalence increases by two- to three-fold, making T2DM one of the most potent risk factors for the progression of MASLD to metabolic dysfunction-associated steatohepatitis (MASH), advanced fibrosis, or cirrhosis (30). Longitudinal data further underscore the clinical implications of this interplay. In a 9-year follow-up study, patients with T2DM and MASLD exhibited a 2.2-fold increase in all-cause mortality compared to those without MASLD (31), highlighting the urgent need for proactive hepatic screening and intervention in this high-risk population.

Currently, imaging modalities such as contrast-enhanced ultrasound, CT, MRS, and liver biopsy are not feasible for large-scale MASLD screening due to their high cost, technical complexity, and limited accessibility. In contrast, demographic, anthropometric, and routine biochemical data are widely available in clinical practice, particularly in primary and secondary healthcare settings. Therefore, there is a clear need for a simple, cost-effective, and non-invasive screening tool based on these readily obtainable indicators to enable early identification of MASLD in patients with T2DM. In response to this need, this study developed a novel DPN tailored for the T2DM population. To promote individualized clinical application, the DPN was further deployed as an interactive web-based calculator and an Excel algorithm tool. To comprehensively evaluate the model's performance, we conducted internal and external validation across multiple datasets. The DPN consistently outperformed existing models—including ZJU, HSI, TyG, and FSI—in the training, validation, and test sets, with all AUC values exceeding 0.75 and all comparisons reaching statistical significance (*P* < 0.05). Moreover, DPN demonstrated significant incremental predictive value, as reflected by positive NRI and IDI (all *P* < 0.05). Calibration plots, DCA, and CIC uniformly demonstrated good model calibration, high clinical net benefit, and broad applicability. Subgroup analyses across stratified populations with T2DM further confirmed the robustness, reliability, and generalizability of the DPN.

Previous studies, both domestic and international, have primarily focused on developing predictive models for fatty liver disease (formerly NAFLD) in general adult populations. Representative examples included the ZJU Index in China (17), the HSI in South Korea (32), and the FSI in the United States (16). In addition, the TyG index has been increasingly recognized as a useful surrogate marker for assessing metabolic risk and disease progression in MASLD (33). Some recent studies have further incorporated machine learning algorithms to enhance predictive accuracy and computational efficiency (34). However, the applicability and predictive performance of these models in individuals with T2DM remain uncertain. Moreover, there is a paucity of screening tools specifically tailored for the T2DM population. Xue et al. (35) and Zhang et al. (36) both developed MASLD risk prediction models using logistic regression in T2DM cohorts. While these models demonstrated good discriminative ability in internal validation (AUCs of 0.756 and 0.848 in training sets; 0.755 and 0.809 in validation sets), several limitations were noted. These include the inclusion of a large number of predictors, lack of external validation in independent populations, absence of head-to-head model comparisons, and limited practical utility due to complex variable requirements. In contrast, the present study addresses these limitations by developing and validating a simplified eight-variable DPN, which demonstrated superior predictive performance compared to existing models across three independent datasets. Additionally, the model was implemented as an interactive web-based calculator and an Excel algorithm tool, enhancing its accessibility and clinical utility. Collectively, these findings highlight the practicality, scalability, and robustness of the DPN as a screening tool for MASLD in populations with T2DM.

Among 27 candidate variables, eight were ultimately included in the model (including gender, age, disease duration, BMI, TG, HDL-C, ALT, and ALB) based on LASSO regression and stepwise AIC selection. Gender has long been recognized as a determinant of susceptibility to metabolic diseases, including T2DM, CVD, hypertension, and MASLD (37). Women with T2DM exhibit a significantly higher risk of CVD than men, with carotid stenosis and ischaemic stroke more prevalent in female patients with metabolic syndrome (38). MASLD, as the hepatic manifestation of metabolic syndrome, also appears to disproportionately affect women with T2DM. A recent study showed that women with prediabetes or T2DM had a higher MASLD risk than men (39), a finding supported by Korean data indicating increased MASLD prevalence in women with elevated fasting glucose or antidiabetic treatment (40). This may be attributed to greater metabolic dysfunction in women, reflected in higher visceral adiposity, dyslipidemia, homeostatic model assessment of insulin resistance (HOMA-IR), and inflammatory markers (e.g., hsCRP), all of which promote hepatic IR and inflammation—key drivers of MASLD pathogenesis (41). Consistent with these findings, this model identified female as an independent risk factor for MASLD in patients with T2DM.

In this study, T2DM patients with MASLD were younger and had a shorter disease duration. The findings of Zhang et al. (36) and Xue et al. (35) were consistent with ours. This suggested that T2DM patients with MASLD were younger compared to those without MASLD. Data indicate that the prevalence of MASLD is often higher in middle-aged populations than in older populations, which may be due to factors such as high work stress, poor lifestyle habits (e.g., irregular diet and lack of exercise), and the widespread presence of metabolic syndrome.

BMI is a widely used anthropometric measure for evaluating obesity and predicting the risk of obesity-related complications, including diabetes, dyslipidaemia, MASLD, and CVD (42). Cohort studies have demonstrated that both central and general obesity are strongly associated with MASLD in T2DM patients, with BMI independently linked to MASLD development (43). Interventions such as dietary modification, pharmacotherapy, and bariatric surgery aim to reduce hepatic steatosis by improving BMI in patients with T2DM or MASLD (44–46). In this study, BMI emerged as a key predictor of MASLD, with significantly higher values observed in affected individuals. These findings underscore the clinical value of BMI in MASLD risk stratification and highlight the importance of weight management in preventing hepatic complications in T2DM.

TG and HDL-C are routinely assessed lipid markers in T2DM management and were included as key predictors in the DPN. Elevated TG and reduced HDL-C levels have been consistently associated with increased risk of T2DM and prediabetes (47), and contribute to the development and progression of MASLD (48). Verwer et al. (49) reported that hepatic steatosis in T2DM was closely linked to postprandial hypertriglyceridaemia, which led to TG enrichment of HDL particles. Impaired clearance of TG-rich HDL compromises its antioxidant function and promotes endothelial dysfunction. Recent studies have proposed lipid ratios as effective markers for identifying individuals at high risk of T2DM and MASLD (50). Li et al. (51) found that the TG/HDL-C ratio had the highest diagnostic value for MASLD in newly diagnosed T2DM patients, with an AUC of 0.732, sensitivity of 73.8%, and specificity of 60.1%. These findings suggest that these lipid parameters may serve as promising candidates for future model refinement.

ALT, a liver-specific enzyme, is commonly elevated in patients with T2DM and MASLD, reflecting hepatocellular injury (52). In Cen's predictive model, ALT ranked as the second most important predictor of MASLD after TG (53), while Mor et al. (54) also identified ALT as a valuable early marker for MASLD in newly diagnosed T2DM patients. Furthermore, elevated ALT has been linked to increased metabolic activity and IR in this population (55). Consistent with prior studies, our findings supported the inclusion of ALT as a key variable in MASLD risk prediction. ALB, the most abundant plasma protein synthesized by the liver, serves critical roles in antioxidation, anti-inflammation, and molecular transport (56). Although evidence on the relationship between ALB and MASLD in T2DM is limited, prior studies have reported conflicting findings. For example, some associate lower albumin levels with metabolic dysfunction and T2DM risk (57), while others link higher albumin to IR (58). In this study, ALB levels were higher in T2DM patients with MASLD, potentially reflecting the influence of hepatoprotective or renoprotective therapies. This observation warrants further investigation.

The MASLD risk prediction tool developed in this study offers a practical and non-invasive approach for early screening in patients with T2DM. First, due to the marked differences in metabolic profiles and MASLD prevalence between T2DM and healthy populations (60%−70% vs. 25%), a T2DM-specific model is warranted. Moreover, the model incorporates routinely collected demographic, anthropometric, and laboratory parameters, ensuring accessibility and ease of application in clinical settings. Third, across three independent cohorts (training, internal validation, and external testing), the model consistently outperformed existing tools in terms of discrimination, calibration, and clinical utility, demonstrating robust stability and generalizability. Lastly, while liver biopsy remains the diagnostic gold standard, our web-based, non-invasive model offers a feasible alternative for large-scale screening and risk stratification in T2DM management.

Nevertheless, this study has several limitations. (1) Although the study included a large sample size of 4,726 patients, the data were derived from a single source. Future multicenter prospective studies with larger cohorts are needed to further validate the model's performance across diverse populations. (2) Some predictors in the model (e.g., age and diabetes duration) were collected via questionnaires, which may introduce recall bias affecting prediction accuracy. (3) Beyond the eight identified predictors, additional factors including dietary patterns, sleep duration, ethnicity, medication use, and comorbidities may influence hepatic metabolism and should be incorporated in future research. (4) The final model contains relatively numerous variables. Developing more parsimonious models with fewer predictors while maintaining high performance warrants further investigation. (5) This study has a retrospective design, and the ultrasound assessments were conducted jointly by physicians; therefore, inter-observer agreement data could not be provided. Future studies should incorporate formal inter-observer agreement analysis for further validation. (6) It is important to acknowledge that the performance of our prediction model is inherently limited by the diagnostic accuracy of abdominal ultrasound for hepatic steatosis. (7) The Fatty Liver Index (FLI) could not be included in the model comparison due to insufficient WC data in our datasets. (8) This model was developed and validated in hospitalized patients with T2DM, who have fundamentally distinct clinical characteristics from outpatients. Therefore, its applicability is currently limited to inpatient risk stratification, and future validation in outpatient cohorts is warranted to assess generalizability.

## 5 Conclusions

In summary, this study developed and validated a clinically applicable prediction model for assessing MASLD risk in patients with T2DM, presented as an interactive web-based tool. The model demonstrated strong discriminative power, calibration, and clinical utility across multiple cohorts. Owing to its simplicity, accessibility, and generalizability, this tool may facilitate early identification of high-risk individuals, thereby improving risk stratification and guiding timely interventions in the management of T2DM.

## Data Availability

The original contributions presented in the study are included in the article/Supplementary material, further inquiries can be directed to the corresponding authors.
