# Supplementary material for: Establishment and evaluation of a novel practical tool for the screening of metabolic dysfunction-associated steatotic liver disease in patients with type 2 diabetes mellitus
Source: Front Nutr. 2025 Dec 12;12:1692394. doi: 10.3389/fnut.2025.1692394 (PMC12740880; doi:10.3389/fnut.2025.1692394)
Supplement: Supplementary file 1 [file Table_1.docx]

**Table S1.** Basic characteristics of T2DM patients with/without MASLD in the training and validation sets

| **Variables** | **Training set** | | | |  | **Validation set** | | | | ***P3*** |
| --- | --- | --- | --- | --- | --- | --- | --- | --- | --- | --- |
|  | **Overall** | **T2DM with non-MASLD** | **T2DM with MASLD** | ***P1*** |  | **Overall** | **T2DM with non-MASLD** | **T2DM with MASLD** | ***P2*** |  |
|  | **(n=3310)** | **(n=2083)** | **(n=1227)** |  |  | **(n=1416)** | **(n=887)** | **(n=529)** |  |  |
| Age (years) | 59.65±11.86 | 61.51±11.29 | 56.47±12.12 | <0.001 |  | 58.93±11.85 | 60.54±11.34 | 56.23±12.20 | <0.001 | 0.055 |
| Male n, (%) | 2039 (61.6) | 1314 (63.0) | 725 (59.1) | 0.022 |  | 862 (60.9) | 542 (61.1) | 320 (60.5) | 0.819 | 0.639 |
| Height (cm) | 168 (160-173) | 168 (160-173) | 168 (161-173) | 0.166 |  | 168 (160-173) | 168 (160-172) | 168 (162-174) | 0.245 | 0.584 |
| Weight (kg) | 68.70±11.10 | 66.49±10.51 | 72.45±11.08 | <0.001 |  | 68.90±11.43 | 66.49±10.38 | 72.94±11.98 | <0.001 | 0.561 |
| BMI (kg/m2) | 24.53±3.09 | 23.80±2.91 | 25.77±2.98 | <0.001 |  | 24.53±3.17 | 23.76±2.92 | 25.83±3.15 | <0.001 | 0.974 |
| Course (years) | 8 (3-14) | 10 (4-15) | 5 (1-10) | <0.001 |  | 7 (3-13) | 9 (4-14) | 5 (1-10) | <0.001 | 0.173 |
| Smoking n, (%) | 532 (16.1) | 325 (15.6) | 207 (16.9) | 0.337 |  | 223 (15.7) | 130 (14.7) | 93 (17.6) | 0.144 | 0.781 |
| Hypertemsion n, (%) | 1729 (52.2) | 1109 (53.2) | 620 (50.5) | 0.132 |  | 719 (50.8) | 452 (51.0) | 267 (50.5) | 0.860 | 0.358 |
| SBP (mmHg) | 137.19±20.98 | 137.20±21.95 | 137.16±19.23 | 0.948 |  | 137.21±21.18 | 136.67±21.67 | 138.12±20.33 | 0.213 | 0.970 |
| DBP (mmHg) | 82.40±11.55 | 81.31±11.74 | 84.25±10.98 | <0.001 |  | 82.83±11.46 | 81.36±11.29 | 85.31±11.32 | <0.001 | 0.239 |
| FBG (mmol/L) | 10.01 (7.50-14.03) | 9.74 (7.30-13.90) | 10.36 (7.92-14.20) | <0.001 |  | 9.82 (7.34-13.83) | 9.63 (7.26-13.69) | 10.29 (7.52-14.19) | 0.142 | 0.313 |
| TC (mmol/L) | 4.23 (3.54-4.97) | 4.16 (3.45-4.88) | 4.36 (3.71-5.10) | <0.001 |  | 4.16 (3.55-4.88) | 4.07 (3.51-4.74) | 4.30 (3.65-5.07) | <0.001 | 0.111 |
| TG (mmol/L) | 1.66 (1.17-2.43) | 1.48 (1.06-2.14) | 1.99 (1.47-2.97) | <0.001 |  | 1.66 (1.15-2.44) | 1.46 (1.04-2.15) | 2.02 (1.42-3.03) | <0.001 | 0.635 |
| HDL-C (mmol/L) | 1.13 (0.95-1.32) | 1.16 (0.98-1.36) | 1.06 (0.91-1.25) | <0.001 |  | 1.13 (0.95-1.33) | 1.17 (0.99-1.38) | 1.07 (0.91-1.27) | <0.001 | 0.496 |
| LDL-C (mmol/L) | 2.72 (2.09-3.37) | 2.69 (2.04-3.32) | 2.76 (2.17-3.44) | <0.001 |  | 2.63 (2.04-3.26) | 2.58 (2.03-3.17) | 2.69 (2.06-3.37) | 0.035 | 0.006 |
| UA (umol/L) | 311 (257-367) | 304 (254-358) | 322 (266-378) | <0.001 |  | 305 (252-364) | 292 (245-351) | 327 (271-379) | <0.001 | 0.069 |
| ALT (U/L) | 21 (15-31) | 19 (14-27) | 25 (18-37) | <0.001 |  | 21 (15-31) | 19 (14-27) | 26 (19-40) | <0.001 | 0.256 |
| AST (U/L) | 20 (16-25) | 19 (15-24) | 21 (17-27) | <0.001 |  | 20 (16-25) | 19 (16-24) | 21 (17-29) | <0.001 | 0.216 |
| GGT (U/L) | 28 (20-43) | 25 (18-37) | 35 (24-51) | <0.001 |  | 28 (19-43) | 24 (17-36) | 34 (24-56.5) | <0.001 | 0.347 |
| ALP (U/L) | 83 (69-102) | 82 (68-101) | 86 (69-105) | 0.027 |  | 84 (68-102) | 83 (68-101) | 85 (69-104) | 0.122 | 0.955 |
| TP (g/L) | 75 (71.3-78.5) | 74.6 (70.8-78.1) | 75.6 (72.2-79.1) | <0.001 |  | 74.8 (71.3-78.3) | 74.1 (70.5-77.7) | 75.8 (72.4-79.4) | <0.001 | 0.455 |
| ALB (g/L) | 45.1 (42.4-47.5) | 44.5 (41.7-46.9) | 46.1 (43.5-48.2) | <0.001 |  | 45.2 (42.4-47.5) | 44.5 (41.9-46.9) | 45.9 (43.6-48.1) | <0.001 | 0.599 |
| GLB (g/L) | 29.8 (26.6-33.3) | 30.0 (26.8-33.5) | 29.4 (26.1-32.95) | 0.002 |  | 29.6 (26.3-33.0) | 29.5 (26.2-32.9) | 29.7 (26.45-33.3) | 0.456 | 0.167 |
| TBIL (umol/L) | 12.9 (9.7-17.1) | 12.5 (9.3-16.9) | 13.5 (10.3-17.8) | <0.001 |  | 12.8 (9.7-16.8) | 12.4 (9.6-16.3) | 13.5 (10.2-18) | <0.001 | 0.961 |
| DBIL (umol/L) | 4.8 (3.6-6.4) | 4.7 (3.5-6.3) | 5.0 (3.8-6.5) | <0.001 |  | 4.9 (3.7-6.4) | 4.8 (3.4-6.1) | 5 (3.8-6.7) | 0.009 | 0.926 |
| IBIL (umol/L) | 8 (5.6-11.2) | 7.7 (5.4-10.9) | 8.4 (6.1-11.8) | <0.001 |  | 7.9 (5.7-10.9) | 7.6 (5.6-10.3) | 8.4 (6.0-12.0) | <0.001 | 0.932 |
| ZJU | 41.23 (37.41-45.95) | 39.94 (35.99-44.46) | 43.54 (39.66-47.91) | <0.001 |  | 40.53 (36.33-44.93) | 38.97 (35.17 - 43.52) | 43.01 (38.75-47.20) | <0.001 | <0.001 |
| HSI | 36.01 (33.01-39.40) | 34.80 (31.94-37.79) | 38.24 (35.21-41.49) | <0.001 |  | 35.96 (32.83-39.27) | 34.54 (31.96-37.46) | 38.29 (35.71-41.63) | <0.001 | 0.840 |
| TyG | 9.51 (9.02-10.08) | 9.39 (8.86-9.94) | 9.73 (9.28-10.26) | <0.001 |  | 9.51 (8.97-10.04) | 9.35 (8.87-9.91) | 9.73 (9.24-10.25) | <0.001 | 0.359 |
| FSI | -0.63 (-1.34 - 0.22) | -0.91 (-1.56 - -0.16) | -0.12 (-0.83 - 0.77) | <0.001 |  | -0.66 (-1.43 - 0.22) | -1.01 (-1.63 - -0.25) | -0.07 (-0.78 - 0.92) | <0.001 | 0.455 |

Abbreviations: *T2DM* type 2 diabetes mellitus *MASLD* non-alcoholic fatty liver disease, *BMI* body mass index, D*N* diabetic nephropathy, *DSPN* diabetic distal symmetrical polyneuropathy, *DPVD* diabetic peripheral vascular disease, *SBP* systolic blood pressure, *DBP* diastolic blood pressure, *FBG* fasting blood glucose, *TC* total cholesterol, *TG* triglyceride, *HDL-C* high density lipoprotein cholesterol, *LDL-C* low density lipoprotein cholesterol, *UA* uric acid, *ALT* alanine transferase, *AST* aspartate transferase, *GGT* γ-glutamyl transpeptadase, *ALP* alkaline phosphatase, *TP* total protein, *ALB* albumin, *GLB* globulin, *TBIL* total bilirubin, *DBIL* direct bilirubin, *IBIL* indirect bilirubin, *ZJU* ZJU index, *HSI* hepatic steatosis index, *TyG* triglyceride-glucose index, *FSI* Framingham steatosis index

**Table S2.** Identification of potential predictors in the training set through univariate logistic analysis and LASSO regression

| **Variables** | **Univariable logistic analysis** |  |  | **LASSO regression analysis** |  |
| --- | --- | --- | --- | --- | --- |
|  | **OR (95% *CI*)** | ***P*** |  | **Lambda.min= 0.00434** | **Lambda.1se=0.02316** |
| Intercept | **-** | **-** |  | -9.387099 | -6.814080 |
| Sex | 1.183 (1.024-1.367) | 0.023 |  | 0.447561 | 0.092450 |
| Age | 0.964 (0.958-0.970) | 0.000 |  | -0.005518 | -0.004479 |
| BMI | 1.254 (1.221-1.288) | 0.000 |  | 0.185552 | 0.156768 |
| Smoking | 1.098 (0.907-1.328) | 0.338 |  | 0 | 0 |
| Course | 0.929 (0.919-0.940) | 0.000 |  | -0.046042 | -0.038621 |
| Hypertension | 0.897 (0.779-1.033) | 0.132 |  | -0.122739 | 0 |
| DN | 0.992 (0.858-1.146) | 0.911 |  | 0.072627 | 0 |
| DSPN | 0.724 (0.578-0.905) | 0.005 |  | 0.024848 | 0 |
| DPVD | 0.663 (0.570-0.770) | 0.000 |  | 0 | 0 |
| SBP | 1.000 (0.997-1.003) | 0.950 |  | 0 | 0 |
| DBP | 1.022 (1.016-1.029) | 0.000 |  | 0.004294 | 0 |
| FBG | 1.017 (1.001-1.032) | 0.032 |  | 0.008947 | 0 |
| UA | 1.002 (1.001-1.003) | 0.000 |  | 0.000354 | 0 |
| ALT | 1.024 (1.020-1.029) | 0.000 |  | 0.011900 | 0.007958 |
| AST | 1.023 (1.016-1.030) | 0.000 |  | 0 | 0 |
| GGT | 1.006 (1.004-1.008) | 0.000 |  | 0.000573 | 0 |
| ALP | 1.001 (0.999-1.003) | 0.300 |  | 0 | 0 |
| TP | 1.040 (1.027-1.053) | 0.000 |  | 0 | 0 |
| ALB | 1.114 (1.094-1.134) | 0.000 |  | 0.078136 | 0.056474 |
| GLB | 0.977 (0.964-0.991) | 0.001 |  | -0.004149 | 0 |
| TBIL | 1.027 (1.017-1.038) | 0.000 |  | 0.008780 | 0 |
| DBIL | 1.041 (1.013-1.070) | 0.004 |  | 0 | 0 |
| IBIL | 1.037 (1.023-1.052) | 0.000 |  | 0 | 0 |
| TC | 1.173 (1.100-1.251) | 0.000 |  | 0 | 0 |
| TG | 1.366 (1.294-1.442) | 0.000 |  | 0.172795 | 0.144404 |
| HDL | 0.324 (0.252-0.416) | 0.000 |  | -0.612527 | -0.149630 |
| LDL | 1.101 (1.023-1.185) | 0.011 |  | 0.100467 | 0 |

Abbreviations: *LASSO* least absolute shrinkage and selection operator, *OR* odds Ratio, *CI* confidence interval, *BMI* body mass index, D*N* diabetic nephropathy, *DSPN* diabetic distal symmetrical polyneuropathy, *DPVD* diabetic peripheral vascular disease, *SBP* systolic blood pressure, *DBP* diastolic blood pressure, *FBG* fasting blood glucose, *UA* uric acid, *ALT* alanine transferase, *AST* aspartate transferase, *GGT* γ-glutamyl transpeptadase, *ALP* alkaline phosphatase, *TP* total protein, *ALB* albumin, *GLB* globulin, *TBIL* total bilirubin, *DBIL* direct bilirubin, *IBIL* indirect bilirubin, *TC* total cholesterol, *TG* triglyceride, *HDL-C* high density lipoprotein cholesterol, *LDL-C* low density lipoprotein cholesterol, *ZJU* ZJU index, *HSI* hepatic steatosis index, *TyG* triglyceride-glucose index, *FSI* Framingham steatosis index.

**Table S3.** Multivariable logistic regression results with stepwise AIC selection

| Intercept and variables | Estimate | Std. Error | Z value | OR | 95% *CI* | *P* value |
| --- | --- | --- | --- | --- | --- | --- |
|  |  |  |  |  |  |  |
| (Intercept) | -8.649 | 0.698 | -12.387 | 0.000 | 0.000-0.000 | <0.001 |
| Sex | 0.473 | 0.088 | 5.353 | 1.605 | 1.349-1.908 | <0.001 |
| Age | -0.009 | 0.004 | -2.194 | 0.991 | 0.983-0.999 | 0.028 |
| BMI | 0.195 | 0.015 | 13.274 | 1.216 | 1.181-1.251 | <0.001 |
| Course | -0.051 | 0.007 | -7.675 | 0.951 | 0.938-0.963 | <0.001 |
| ALT | 0.013 | 0.002 | 5.569 | 1.013 | 1.009-1.018 | <0.001 |
| ALB | 0.088 | 0.011 | 8.391 | 1.092 | 1.070-1.115 | <0.001 |
| TG | 0.196 | 0.030 | 6.625 | 1.216 | 1.148-1.289 | <0.001 |
| HDL | -0.594 | 0.159 | -3.745 | 0.552 | 0.405-0.753 | <0.001 |

Abbreviations *BMI* body mass index, *ALT* alanine aminotransferase, *ALB* albumin, *TG* triglyceride, *HDL-C* high density lipoprotein, *OR* odd ratio, *CI* confidence interval.

**Table S4**. Alternative cut-off values, sensitivity, and specificity by dataset for DPN

| Dataset | Clinical Scenario | Cut-off Value | Sensitivity | Specificity |
| --- | --- | --- | --- | --- |
| Training Set | Rule-Out (High Sensitivity) | -1.256 | 0.900 | 0.444 |
|  | Rule-In (High Specificity) | 0.243 | 0.374 | 0.900 |
| Validation set | Rule-Out (High Sensitivity) | -1.222 | 0.900 | 0.448 |
|  | Rule-In (High Specificity) | 0.350 | 0.333 | 0.900 |
| NHANES set | Rule-Out (High Sensitivity) | -1.415 | 0.901 | 0.401 |
|  | Rule-In (High Specificity) | 1.125 | 0.314 | 0.905 |

**Table S5** Performance comparison between DPN and other models in predicting MASLD risk among male participants

| Models | AUC (95% CI) | *P*1 values | SEN (95% CI) | SPE (95% CI) | PPV (95% CI) | NPV (95% CI) | PLR (95% CI) | NLR (95% CI) | DOR | Yodan | Cut off values | NRI (95% CI) | *P*2 values | IDI (95% CI) | *P*3 values |
| --- | --- | --- | --- | --- | --- | --- | --- | --- | --- | --- | --- | --- | --- | --- | --- |
| **Male in the training set** | |  |  |  |  |  |  |  |  |  |  |  |  |  |  |
| DPN | 0.789 (0.770-0.809) | - | 0.761 (0.728-0.792) | 0.688 (0.662-0.713) | 0.574 (0.542-0.605) | 0.839 (0.816-0.861) | 2.440 (2.230-2.670) | 0.347 (0.304-0.395) | 7.032 | 0.449 | -0.727 | Ref | - | Ref | - |
| ZJU | 0.680 (0.657-0.703) | <0.001 | 0.677 (0.642-0.711) | 0.596 (0.569-0.622) | 0.480 (0.449-0.512) | 0.770 (0.743-0.795) | 1.676 (1.543-1.820) | 0.542 (0.487-0.602) | 3.092 | 0.273 | 40.785 | 0.234 (0.187-0.278) | <0.001 | 0.147 (0.130-0.167) | <0.001 |
| HSI | 0.725 (0.702-0.747) | <0.001 | 0.757 (0.724-0.788) | 0.574 (0.547-0.601) | 0.495 (0.465-0.525) | 0.811 (0.784-0.835) | 1.777 (1.648-1.915) | 0.423 (0.372-0.482) | 4.201 | 0.331 | 35.325 | 0.112 (0.068-0.155) | <0.001 | 0.091 (0.076-0.106) | <0.001 |
| TyG | 0.642 (0.618-0.667) | <0.001 | 0.756 (0.723-0.786) | 0.461 (0.434-0.489) | 0.436 (0.409-0.464) | 0.774 (0.743-0.802) | 1.403 (1.315-1.497) | 0.529 (0.465-0.603) | 2.652 | 0.217 | 9.262 | 0.258 (0.214-0.303) | <0.001 | 0.175 (0.156-0.193) | <0.001 |
| FSI | 0.713 (0.690-0.735) | <0.001 | 0.683 (0.647-0.716) | 0.639 (0.613-0.665) | 0.511 (0.479-0.543) | 0.785 (0.759-0.809) | 1.893 (1.734-2.066) | 0.496 (0.446-0.553) | 3.817 | 0.322 | -0.481 | 0.157 (0.116-0.199) | <0.001 | 0.118 (0.103-0.133) | <0.001 |
| **Male in the validation set** | |  |  |  |  |  |  |  |  |  |  |  |  |  |  |
| DPN | 0.767 (0.735-0.799) | - | 0.763 (0.711-0.807) | 0.655 (0.613-0.695) | 0.566 (0.518-0.613) | 0.824 (0.784-0.858) | 2.210 (1.938-2.520) | 0.363 (0.297-0.442) | 6.088 | 0.418 | -0.708 | Ref | - | Ref | - |
| ZJU | 0.669 (0.633-0.706) | <0.001 | 0.550 (0.494-0.605) | 0.703 (0.662-0.741) | 0.522 (0.468-0.576) | 0.726 (0.685-0.763) | 1.852 (1.573-2.180) | 0.640 (0.566-0.724) | 2.894 | 0.253 | 42.965 | 0.171 (0.101-0.240) | <0.001 | 0.114 (0.091-0.138) | <0.001 |
| HSI | 0.742 (0.709-0.775) | 0.039 | 0.778 (0.728-0.822) | 0.616 (0.574-0.657) | 0.545 (0.498-0.591) | 0.825 (0.783-0.860) | 2.028 (1.795-2.290) | 0.360 (0.293-0.443) | 5.633 | 0.394 | 35.575 | 0.061 (0.002-0.120) | 0.044 | 0.045 (0.025-0.064) | <0.001 |
| TyG | 0.629 (0.591-0.668) | <0.001 | 0.656 (0.601-0.708) | 0.557 (0.514-0.599) | 0.467 (0.420-0.514) | 0.733 (0.687-0.775) | 1.482 (1.310-1.677) | 0.617 (0.529-0.720) | 2.402 | 0.213 | 9.460 | 0.228 (0.159-0.298) | <0.001 | 0.148 (0.121-0.174) | <0.001 |
| FSI | 0.729 (0.696-0.763) | 0.006 | 0.625 (0.569-0.678) | 0.729 (0.689-0.765) | 0.576 (0.522-0.629) | 0.767 (0.728-0.802) | 2.304 (1.960-2.710) | 0.515 (0.446-0.594) | 4.474 | 0.354 | -0.218 | 0.125 (0.063-0.187) | <0.001 | 0.082 (0.061-0.103) | <0.001 |

Abbreviations: *DPN* diagnostic predictive nomogram, *ZJU* the ZJU index, *HSI* hepatic steatosis index, *TyG* triglycerideglucose index, *FSI* Framingham steatosis index, *AUC* area under the curve, *SEN* sensitivity, *SPE* specificity, *PPV* positive predictive value, *NPV* negative predictive value, *PLR* positive likelihood ratio, *NLR* negative likelihood ratio, *DOR* diagnostic odds ratio, *NRI* net reclassifcation index, *IDI* integrated discrimination improvement

**Table S6** Performance comparison between DPN and other models in predicting MASLD risk among female participants

| Models | AUC (95% CI) | *P*1 values | SEN (95% CI) | SPE (95% CI) | PPV (95% CI) | NPV (95% CI) | PLR (95% CI) | NLR (95% CI) | DOR | Yodan | Cut off values | NRI (95% CI) | *P*2 values | IDI (95% CI) | *P*3 values |
| --- | --- | --- | --- | --- | --- | --- | --- | --- | --- | --- | --- | --- | --- | --- | --- |
| **Female in the training set** | | | | | | | | | | | | | | | |
| DPN | 0.750 (0.723-0.777) | - | 0.795 (0.756-0.829) | 0.588 (0.552-0.623) | 0.557 (0.520-0.594) | 0.814 (0.779-0.845) | 1.928 (1.753-2.121) | 0.349 (0.293-0.415) | 5.524 | 0.383 | -0.756 | Ref | - | Ref | - |
| ZJU | 0.640 (0.610-0.670) | <0.001 | 0.707 (0.665-0.746) | 0.508 (0.473-0.544) | 0.484 (0.448-0.521) | 0.727 (0.687-0.764) | 1.439 (1.313-1.576) | 0.576 (0.501-0.661) | 2.498 | 0.215 | 40.775 | 0.247 (0.189-0.306) | <0.001 | 0.124 (0.105-0.143) | <0.001 |
| HSI | 0.688 (0.658-0.717) | <0.001 | 0.528 (0.483-0.572) | 0.754 (0.722-0.784) | 0.584 (0.537-0.629) | 0.710 (0.677-0.741) | 2.148 (1.851-2.493) | 0.626 (0.570-0.687) | 3.431 | 0.282 | 37.885 | 0.128 (0.079-0.176) | <0.001 | 0.082 (0.066-0.098) | <0.001 |
| TyG | 0.621 (0.591-0.652) | <0.001 | 0.765 (0.725-0.801) | 0.414 (0.379-0.450) | 0.460 (0.426-0.494) | 0.729 (0.685-0.770) | 1.304 (1.208-1.408) | 0.568 (0.484-0.668) | 2.296 | 0.179 | 9.269 | 0.242 (0.181-0.303) | <0.001 | 0.136 (0.115-0.157) | <0.001 |
| FSI | 0.677 (0.648-0.707) | <0.001 | 0.643 (0.600-0.685) | 0.629 (0.594-0.663) | 0.531 (0.491-0.571) | 0.730 (0.694-0.763) | 1.736 (1.551-1.943) | 0.567 (0.503-0.638) | 3.062 | 0.272 | -0.680 | 0.136 (0.083-0.189) | <0.001 | 0.095 (0.079-0.111) | <0.001 |
| **Female in the validation set** | | | | | | | | | | | | | | | |
| DPN | 0.767 (0.727-0.806) | - | 0.775 (0.711-0.829) | 0.670 (0.617-0.718) | 0.587 (0.526-0.645) | 0.831 (0.780-0.872) | 2.346 (1.985-2.772) | 0.336 (0.260-0.433) | 6.982 | 0.445 | -0.719 | Ref | - | Ref | - |
| ZJU | 0.668 (0.622-0.713) | <0.001 | 0.646 (0.577-0.710) | 0.629 (0.575-0.680) | 0.513 (0.451-0.575) | 0.746 (0.691-0.794) | 1.741 (1.469-2.064) | 0.563 (0.467-0.678) | 3.092 | 0.275 | 40.155 | 0.192 (0.103-0.281) | <0.001 | 0.123 (0.092-0.153) | <0.001 |
| HSI | 0.716 (0.673-0.759) | 0.007 | 0.737 (0.671-0.794) | 0.617 (0.564-0.669) | 0.538 (0.479-0.597) | 0.795 (0.740-0.840) | 1.926 (1.647-2.252) | 0.426 (0.339-0.537) | 4.511 | 0.354 | 35.745 | 0.104 (0.024-0.184) | 0.011 | 0.078 (0.049-0.106) | <0.001 |
| TyG | 0.642 (0.596-0.688) | <0.001 | 0.641 (0.572-0.705) | 0.580 (0.526-0.632) | 0.480 (0.421-0.541) | 0.727 (0.670-0.778) | 1.525 (1.300-1.790) | 0.619 (0.514-0.745) | 2.464 | 0.221 | 9.502 | 0.221 (0.133-0.308) | <0.001 | 0.141 (0.108-0.173) | <0.001 |
| FSI | 0.708 (0.664-0.752) | 0.001 | 0.718 (0.651-0.777) | 0.626 (0.572-0.677) | 0.538 (0.477-0.597) | 0.785 (0.731-0.832) | 1.919 (1.634-2.254) | 0.451 (0.362-0.562) | 4.255 | 0.344 | -0.803 | 0.090 (0.011-0.168) | 0.026 | 0.077 (0.051-0.103) | <0.001 |

Abbreviations: *DPN* diagnostic predictive nomogram, *ZJU* the ZJU index, *HSI* hepatic steatosis index, *TyG* triglycerideglucose index, *FSI* Framingham steatosis index, *AUC* area under the curve, *SEN* sensitivity, *SPE* specificity, *PPV* positive predictive value, *NPV* negative predictive value, *PLR* positive likelihood ratio, *NLR* negative likelihood ratio, *DOR* diagnostic odds ratio, *NRI* net reclassifcation index, *IDI* integrated discrimination improvement

**Table S7** Performance comparison between DPN and other models in predicting MASLD risk among participants aged <60 years

| Models | AUC (95% CI) | P1 values | SEN (95% CI) | SPE (95% CI) | PPV (95% CI) | NPV (95% CI) | PLR (95% CI) | NLR (95% CI) | DOR | Yodan | Cut off values | NRI (95% CI) | P2 values | IDI (95% CI) | P3 values |
| --- | --- | --- | --- | --- | --- | --- | --- | --- | --- | --- | --- | --- | --- | --- | --- |
| **Age<60 in the training set** | | | | | | | | | | | | | | | |
| DPN | 0.766 (0.743-0.789) | - | 0.754 (0.721-0.785) | 0.648 (0.616-0.679) | 0.633 (0.600-0.665) | 0.766 (0.734-0.795) | 2.143 (1.943-2.364) | 0.379 (0.334-0.431) | 5.654 | 0.402 | -0.397 | Ref | - | Ref | - |
| ZJU | 0.660 (0.634-0.686) | <0.001 | 0.616 (0.579-0.651) | 0.627 (0.594-0.659) | 0.571 (0.535-0.606) | 0.669 (0.636-0.701) | 1.651 (1.491-1.829) | 0.612 (0.558-0.673) | 2.698 | 0.243 | 42.425 | 0.178 (0.123-0.233) | <0.001 | 0.130 (0.113-0.147) | <0.001 |
| HSI | 0.708 (0.683-0.733) | <0.001 | 0.729 (0.695-0.761) | 0.582 (0.549-0.615) | 0.585 (0.552-0.617) | 0.727 (0.693-0.759) | 1.746 (1.598-1.909) | 0.465 (0.412-0.525) | 3.755 | 0.311 | 36.535 | 0.074 (0.027-0.121) | 0.002 | 0.083 (0.068-0.098) | <0.001 |
| TyG | 0.614 (0.587-0.641) | <0.001 | 0.540 (0.503-0.577) | 0.622 (0.590-0.654) | 0.536 (0.499-0.572) | 0.627 (0.594-0.658) | 1.431 (1.285-1.593) | 0.739 (0.682-0.801) | 1.936 | 0.162 | 9.708 | 0.232 (0.176-0.288) | <0.001 | 0.164 (0.145-0.183) | <0.001 |
| FSI | 0.698 (0.673-0.723) | <0.001 | 0.667 (0.631-0.701) | 0.621 (0.589-0.653) | 0.587 (0.552-0.621) | 0.698 (0.665-0.730) | 1.762 (1.597-1.944) | 0.536 (0.483-0.595) | 3.287 | 0.288 | -0.512 | 0.100 (0.052-0.147) | <0.001 | 0.103 (0.088-0.118) | <0.001 |
| **Age<60 in the validation set** | | | | | | | | | | | | | | | |
| DPN | 0.767 (0.733-0.801) | - | 0.853 (0.808-0.889) | 0.569 (0.520-0.618) | 0.601 (0.554-0.647) | 0.836 (0.786-0.876) | 1.981 (1.756-2.234) | 0.258 (0.197-0.338) | 7.678 | 0.422 | -0.623 | Ref | - | Ref | - |
| ZJU | 0.679 (0.640-0.717) | <0.001 | 0.773 (0.722-0.818) | 0.513 (0.464-0.563) | 0.548 (0.500-0.594) | 0.748 (0.693-0.797) | 1.589 (1.415-1.784) | 0.442 (0.358-0.545) | 3.595 | 0.286 | 39.855 | 0.134 (0.057-0.211) | 0.001 | 0.111 (0.086-0.137) | <0.001 |
| HSI | 0.747 (0.712-0.782) | 0.117 | 0.799 (0.749-0.841) | 0.591 (0.542-0.639) | 0.598 (0.549-0.645) | 0.794 (0.744-0.837) | 1.954 (1.718-2.223) | 0.340 (0.272-0.426) | 5.747 | 0.390 | 36.335 | 0.014 (-0.054-0.082) | 0.680 | 0.037 (0.014-0.059) | 0.001 |
| TyG | 0.644 (0.604-0.684) | <0.001 | 0.668 (0.612-0.719) | 0.564 (0.515-0.613) | 0.539 (0.488-0.589) | 0.690 (0.638-0.739) | 1.533 (1.340-1.755) | 0.589 (0.501-0.691) | 2.603 | 0.232 | 9.533 | 0.207 (0.124-0.290) | <0.001 | 0.142 (0.114-0.171) | <0.001 |
| FSI | 0.744 (0.708-0.779) | 0.078 | 0.799 (0.749-0.841) | 0.582 (0.532-0.629) | 0.592 (0.544-0.639) | 0.791 (0.740-0.835) | 1.909 (1.681-2.167) | 0.346 (0.277-0.433) | 5.517 | 0.381 | -0.812 | 0.043 (-0.029-0.114) | 0.242 | 0.060 (0.039-0.082) | <0.001 |

Abbreviations: *DPN* diagnostic predictive nomogram, *ZJU* the ZJU index, *HSI* hepatic steatosis index, *TyG* triglycerideglucose index, *FSI* Framingham steatosis index, *AUC* area under the curve, *SEN* sensitivity, *SPE* specificity, *PPV* positive predictive value, *NPV* negative predictive value, *PLR* positive likelihood ratio, *NLR* negative likelihood ratio, *DOR* diagnostic odds ratio, *NRI* net reclassifcation index, *IDI* integrated discrimination improvement

**Table S8** Performance comparison between DPN and other models in predicting MASLD risk among participants aged ≥ 60 years

| Models | AUC (95% CI) | P1 values | SEN (95% CI) | SPE (95% CI) | PPV (95% CI) | NPV (95% CI) | PLR (95% CI) | NLR (95% CI) | DOR | Yodan | Cut off values | NRI (95% CI) | P2 values | IDI (95% CI) | P3 values |
| --- | --- | --- | --- | --- | --- | --- | --- | --- | --- | --- | --- | --- | --- | --- | --- |
| **Age≥60 in the training set** | | | | | | | | | | | | | | | |
| DPN | 0.764 (0.741-0.788) | - | 0.726 (0.684-0.764) | 0.682 (0.654-0.708) | 0.492 (0.455-0.529) | 0.854 (0.830-0.876) | 2.281 (2.066-2.519) | 0.402 (0.349-0.464) | 5.674 | 0.408 | -0.942 | Ref | - | Ref | - |
| ZJU | 0.657 (0.630-0.684) | <0.001 | 0.805 (0.767-0.838) | 0.446 (0.418-0.475) | 0.382 (0.352-0.412) | 0.844 (0.812-0.871) | 1.454 (1.360-1.555) | 0.436 (0.365-0.522) | 3.335 | 0.251 | 38.440 | 0.212 (0.167-0.257) | <0.001 | 0.128 (0.110-0.146) | <0.001 |
| HSI | 0.688 (0.661-0.715) | <0.001 | 0.730 (0.688-0.768) | 0.550 (0.521-0.579) | 0.408 (0.376-0.441) | 0.827 (0.799-0.853) | 1.622 (1.494-1.762) | 0.491 (0.425-0.568) | 3.303 | 0.280 | 34.775 | 0.151 (0.106-0.195) | <0.001 | 0.090 (0.074-0.107) | <0.001 |
| TyG | 0.644 (0.617-0.672) | <0.001 | 0.755 (0.715-0.792) | 0.487 (0.458-0.516) | 0.385 (0.354-0.416) | 0.824 (0.794-0.851) | 1.472 (1.367-1.587) | 0.502 (0.430-0.587) | 2.932 | 0.242 | 9.272 | 0.234 (0.190-0.279) | <0.001 | 0.134 (0.115-0.153) | <0.001 |
| FSI | 0.687 (0.660-0.714) | <0.001 | 0.694 (0.651-0.733) | 0.611 (0.582-0.639) | 0.431 (0.397-0.466) | 0.825 (0.797-0.849) | 1.784 (1.627-1.955) | 0.501 (0.439-0.572) | 3.561 | 0.305 | -0.672 | 0.155 (0.112-0.198) | <0.001 | 0.102 (0.086-0.118) | <0.001 |
| **Age≥60 in the validation set** | | | | | | | | | | | | | | | |
| DPN | 0.757 (0.720-0.794) | - | 0.759 (0.696-0.814) | 0.651 (0.606-0.694) | 0.497 (0.442-0.552) | 0.856 (0.815-0.890) | 2.178 (1.885-2.514) | 0.370 (0.291-0.469) | 5.886 | 0.410 | -1.024 | Ref | - | Ref | - |
| ZJU | 0.634 (0.591-0.678) | <0.001 | 0.694 (0.628-0.754) | 0.513 (0.467-0.558) | 0.393 (0.344-0.444) | 0.787 (0.736-0.830) | 1.425 (1.254-1.619) | 0.596 (0.486-0.732) | 2.391 | 0.207 | 38.415 | 0.165 (0.093-0.237) | <0.001 | 0.109 (0.084-0.134) | <0.001 |
| HSI | 0.697 (0.656-0.738) | 0.001 | 0.657 (0.589-0.720) | 0.679 (0.634-0.720) | 0.481 (0.423-0.540) | 0.814 (0.771-0.850) | 2.045 (1.740-2.405) | 0.505 (0.419-0.608) | 4.050 | 0.336 | 35.575 | 0.111 (0.045-0.177) | <0.001 | 0.070 (0.050-0.092) | <0.001 |
| TyG | 0.606 (0.562-0.651) | <0.001 | 0.579 (0.510-0.645) | 0.590 (0.545-0.635) | 0.391 (0.337-0.447) | 0.755 (0.708-0.798) | 1.413 (1.208-1.652) | 0.714 (0.609-0.837) | 1.979 | 0.169 | 9.459 | 0.166 (0.096-0.236) | <0.001 | 0.125 (0.098-0.152) | <0.001 |
| FSI | 0.678 (0.636-0.720) | <0.001 | 0.713 (0.647-0.771) | 0.574 (0.528-0.618) | 0.431 (0.380-0.485) | 0.815 (0.768-0.854) | 1.672 (1.462-1.912) | 0.500 (0.404-0.619) | 3.344 | 0.287 | -0.799 | 0.115 (0.050-0.181) | 0.001 | 0.088 (0.065-0.111) | <0.001 |

Abbreviations: *DPN* diagnostic predictive nomogram, *ZJU* the ZJU index, *HSI* hepatic steatosis index, *TyG* triglycerideglucose index, *FSI* Framingham steatosis index, *AUC* area under the curve, *SEN* sensitivity, *SPE* specificity, *PPV* positive predictive value, *NPV* negative predictive value, *PLR* positive likelihood ratio, *NLR* negative likelihood ratio, *DOR* diagnostic odds ratio, *NRI* net reclassifcation index, *IDI* integrated discrimination improvement

**Table S9** Performance comparison between DPN and existing models in predicting MASLD risk among participants with disease duration < 20 years

| Models | AUC (95% CI) | P1 values | SEN (95% CI) | SPE (95% CI) | PPV (95% CI) | NPV (95% CI) | PLR (95% CI) | NLR (95% CI) | DOR | Yodan | Cut off values | NRI (95% CI) | P2 values | IDI (95% CI) | P3 values |
| --- | --- | --- | --- | --- | --- | --- | --- | --- | --- | --- | --- | --- | --- | --- | --- |
| **Course<20 in the training set** | | | | | | | | | | | | | | | |
| DPN | 0.766 (0.749-0.783) | - | 0.814 (0.790-0.836) | 0.586 (0.563-0.609) | 0.561 (0.537-0.585) | 0.829 (0.807-0.849) | 1.967 (1.849-2.092) | 0.317 (0.281-0.358) | 6.205 | 0.400 | -0.761 | Ref | - | Ref | - |
| ZJU | 0.659 (0.640-0.679) | <0.001 | 0.685 (0.657-0.711) | 0.558 (0.534-0.581) | 0.502 (0.477-0.527) | 0.731 (0.707-0.754) | 1.549 (1.451-1.653) | 0.565 (0.519-0.616) | 2.742 | 0.243 | 40.775 | 0.206 (0.168-0.244) | <0.001 | 0.128 (0.116-0.141) | <0.001 |
| HSI | 0.707 (0.688-0.726) | <0.001 | 0.664 (0.636-0.691) | 0.643 (0.620-0.665) | 0.548 (0.521-0.574) | 0.746 (0.724-0.768) | 1.862 (1.728-2.006) | 0.522 (0.481-0.567) | 3.567 | 0.307 | 36.525 | 0.079 (0.045-0.113) | <0.001 | 0.076 (0.065-0.087) | <0.001 |
| TyG | 0.625 (0.604-0.645) | <0.001 | 0.752 (0.726-0.777) | 0.436 (0.413-0.459) | 0.465 (0.442-0.487) | 0.730 (0.702-0.756) | 1.334 (1.265-1.405) | 0.569 (0.513-0.629) | 2.344 | 0.188 | 9.268 | 0.229 (0.190-0.267) | <0.001 | 0.153 (0.139-0.167) | <0.001 |
| FSI | 0.690 (0.671-0.710) | <0.001 | 0.701 (0.674-0.727) | 0.584 (0.561-0.607) | 0.523 (0.498-0.548) | 0.750 (0.727-0.773) | 1.687 (1.578-1.803) | 0.511 (0.468-0.559) | 3.301 | 0.285 | -0.680 | 0.137 (0.102-0.172) | <0.001 | 0.103 (0.092-0.115) | <0.001 |
| **Course<20 in the validation set** | | | | | | | | | | | | | | | |
| DPN | 0.756 (0.729-0.782) | - | 0.788 (0.749-0.823) | 0.618 (0.583-0.652) | 0.571 (0.533-0.608) | 0.819 (0.785-0.849) | 2.065 (1.868-2.283) | 0.342 (0.289-0.406) | 6.038 | 0.406 | -0.711 | Ref | - | Ref | - |
| ZJU | 0.664 (0.634-0.694) | <0.001 | 0.655 (0.611-0.696) | 0.599 (0.563-0.633) | 0.513 (0.473-0.552) | 0.729 (0.693-0.763) | 1.633 (1.467-1.817) | 0.577 (0.510-0.651) | 2.830 | 0.254 | 40.425 | 0.137 (0.078-0.196) | <0.001 | 0.103 (0.084-0.121) | <0.001 |
| HSI | 0.728 (0.700-0.728) | 0.010 | 0.764 (0.724-0.800) | 0.602 (0.566-0.636) | 0.553 (0.515-0.590) | 0.799 (0.763-0.830) | 1.919 (1.738-2.118) | 0.392 (0.334-0.459) | 4.895 | 0.366 | 35.605 | 0.052 (0.002-0.103) | 0.044 | 0.044 (0.028-0.059) | <0.001 |
| TyG | 0.632 (0.601-0.663) | <0.001 | 0.649 (0.605-0.690) | 0.569 (0.534-0.604) | 0.492 (0.454-0.531) | 0.716 (0.678-0.751) | 1.507 (1.359-1.671) | 0.617 (0.547-0.696) | 2.442 | 0.218 | 9.460 | 0.194 (0.134-0.253) | <0.001 | 0.130 (0.109-0.151) | <0.001 |
| FSI | 0.717 (0.689-0.745) | 0.001 | 0.758 (0.718-0.795) | 0.577 (0.541-0.612) | 0.536 (0.498-0.573) | 0.788 (0.751-0.820) | 1.794 (1.630-1.974) | 0.418 (0.358-0.490) | 4.292 | 0.335 | -0.812 | 0.094 (0.042-0.145) | <0.001 | 0.070 (0.054-0.086) | <0.001 |

Abbreviations: *DPN* diagnostic predictive nomogram, *ZJU* the ZJU index, *HSI* hepatic steatosis index, *TyG* triglycerideglucose index, *FSI* Framingham steatosis index, *AUC* area under the curve, *SEN* sensitivity, *SPE* specificity, *PPV* positive predictive value, *NPV* negative predictive value, *PLR* positive likelihood ratio, *NLR* negative likelihood ratio, *DOR* diagnostic odds ratio, *NRI* net reclassifcation index, *IDI* integrated discrimination improvement

**Table S10** Performance comparison between DPN and existing models in predicting MASLD risk among participants with disease duration ≥ 20 years

| Models | AUC (95% CI) | P1 values | SEN (95% CI) | SPE (95% CI) | PPV (95% CI) | NPV (95% CI) | PLR (95% CI) | NLR (95% CI) | DOR | Yodan | Cut off values | NRI (95% CI) | P2 values | IDI (95% CI) | P3 values |
| --- | --- | --- | --- | --- | --- | --- | --- | --- | --- | --- | --- | --- | --- | --- | --- |
| **Course≥20 in the training set** | | | | | | | | | | | | | | | |
| DPN | 0.764 (0.703-0.824) | - | 0.754 (0.633-0.846) | 0.653 (0.597-0.706) | 0.331 (0.259-0.411) | 0.921 (0.874-0.952) | 2.175 (1.771-2.670) | 0.377 (0.249-0.572) | 5.769 | 0.407 | -1.749 | Ref | - | Ref | - |
| ZJU | 0.725 (0.664-0.786) | 0.013 | 0.739 (0.617-0.834) | 0.637 (0.580-0.691) | 0.317 (0.247-0.395) | 0.915 (0.866-0.947) | 2.036 (1.659-2.498) | 0.410 (0.274-0.612) | 4.966 | 0.376 | 41.185 | 0.135 (0.041-0.229) | 0.005 | 0.113 (0.064-0.163) | <0.001 |
| HSI | 0.683 (0.618-0.747) | 0.001 | 0.754 (0.633-0.846) | 0.607 (0.550-0.662) | 0.304 (0.237-0.380) | 0.915 (0.866-0.948) | 1.919 (1.580-2.331) | 0.406 (0.267-0.616) | 4.727 | 0.361 | 34.770 | 0.183 (0.087-0.280) | <0.001 | 0.151 (0.101-0.201) | <0.001 |
| TyG | 0.748 (0.688-0.808) | 0.059 | 0.681 (0.557-0.785) | 0.743 (0.689-0.790) | 0.376 (0.292-0.467) | 0.911 (0.866-0.942) | 2.646 (2.060-3.398) | 0.429 (0.303-0.608) | 6.168 | 0.424 | 9.758 | 0.159 (0.054-0.265) | 0.003 | 0.087 (0.040-0.134) | <0.001 |
| FSI | 0.758 (0.698-0.817) | 0.088 | 0.768 (0.648-0.858) | 0.686 (0.630-0.738) | 0.358 (0.282-0.441) | 0.929 (0.884-0.957) | 2.450 (1.984-3.026) | 0.338 (0.219-0.521) | 7.249 | 0.454 | -0.566 | 0.145 (0.051-0.239) | 0.003 | 0.095 (0.055-0.134) | <0.001 |
| **Course≥20 in the validation set** | | | | | | | | | | | | | | | |
| DPN | 0.804 (0.700-0.908) | - | 0.679 (0.476-0.834) | 0.862 (0.780-0.918) | 0.559 (0.381-0.724) | 0.913 (0.836-0.957) | 4.931 (2.889-8.416) | 0.373 (0.217-0.640) | 13.220 | 0.541 | -1.255 | Ref | - | Ref | - |
| ZJU | 0.729 (0.639-0.819) | 0.002 | 1.000 (0.850-1.000) | 0.440 (0.346-0.539) | 0.315 (0.223-0.423) | 1.000 (0.908-1.000) | 1.787 (1.513-2.111) | 0 (0-NaN) | - | 0.440 | 38.720 | 0.277 (0.101-0.452) | 0.002 | 0.191 (0.110-0.271) | <0.001 |
| HSI | 0.759 (0.657-0.861) | 0.025 | 0.679 (0.476-0.834) | 0.807 (0.718-0.874) | 0.475 (0.318-0.637) | 0.907 (0.827-0.954) | 3.522 (2.221-5.586) | 0.398 (0.231-0.685) | 8.849 | 0.486 | 37.010 | 0.250 (0.031-0.469) | 0.025 | 0.166 (0.062-0.270) | 0.002 |
| TyG | 0.673 (0.566-0.779) | <0.001 | 1.000 (0.850-1.000) | 0.275 (0.196-0.370) | 0.262 (0.184-0.357) | 1.000 (0.859-1.000) | 1.380 (1.230-1.549) | 0 (0-NaN) | - | 0.275 | 8.931 | 0.249 (0.076-0.422) | 0.005 | 0.208 (0.134-0.281) | <0.001 |
| FSI | 0.783 (0.701-0.866) | 0.016 | 0.964 (0.798-0.998) | 0.523 (0.426-0.619) | 0.342 (0.241-0.458) | 0.983 (0.895-0.999) | 2.021 (1.640-2.491) | 0.068 (0.010-0.476) | 29.721 | 0.487 | -0.888 | 0.188 (0.007-0.369) | 0.042 | 0.116 (0.050-0.183) | 0.001 |

Abbreviations: *DPN* diagnostic predictive nomogram, *ZJU* the ZJU index, *HSI* hepatic steatosis index, *TyG* triglycerideglucose index, *FSI* Framingham steatosis index, *AUC* area under the curve, *SEN* sensitivity, *SPE* specificity, *PPV* positive predictive value, *NPV* negative predictive value, *PLR* positive likelihood ratio, *NLR* negative likelihood ratio, *DOR* diagnostic odds ratio, *NRI* net reclassifcation index, *IDI* integrated discrimination improvement

**Table S11** Performance comparison between DPN and other models in predicting MASLD risk among patients with hypertension

| Models | AUC (95% CI) | *P*1 values | SEN (95% CI) | SPE (95% CI) | PPV (95% CI) | NPV (95% CI) | PLR (95% CI) | NLR (95% CI) | DOR | Yodan | Cut off values | NRI (95% CI) | *P*2 values | IDI (95% CI) | *P*3 values |
| --- | --- | --- | --- | --- | --- | --- | --- | --- | --- | --- | --- | --- | --- | --- | --- |
| **Hypertension in the training set** | | | | | | | | | | | | | | | |
| DPN | 0.779 (0.757-0.801) | - | 0.787 (0.752-0.818) | 0.646 (0.617-0.674) | 0.554 (0.520-0.587) | 0.844 (0.818-0.868) | 2.221 (2.031-2.429) | 0.330 (0.283-0.384) | 6.730 | 0.433 | -0.761 | Ref | - | Ref | - |
| ZJU | 0.661 (0.635-0.687) | <0.001 | 0.727 (0.690-0.762) | 0.530 (0.500-0.560) | 0.464 (0.432-0.496) | 0.777 (0.745-0.806) | 1.548 (1.431-1.676) | 0.514 (0.451-0.586) | 3.012 | 0.257 | 40.695 | 0.258 (0.209-0.306) | <0.001 | 0.151 (0.133-0.169) | <0.001 |
| HSI | 0.706 (0.682-0.731) | <0.001 | 0.716 (0.679-0.751) | 0.586 (0.556-0.615) | 0.492 (0.459-0.525) | 0.787 (0.757-0.814) | 1.730 (1.588-1.885) | 0.484 (0.427-0.550) | 3.574 | 0.302 | 36.015 | 0.138 (0.095-0.181) | <0.001 | 0.100 (0.084-0.115) | <0.001 |
| TyG | 0.622 (0.595-0.649) | <0.001 | 0.745 (0.709-0.779) | 0.441 (0.412-0.471) | 0.427 (0.397-0.457) | 0.756 (0.720-0.788) | 1.333 (1.243-1.429) | 0.578 (0.504-0.663) | 2.306 | 0.186 | 9.295 | 0.287 (0.239-0.335) | <0.001 | 0.176 (0.156-0.196) | <0.001 |
| FSI | 0.705 (0.680-0.730) | <0.001 | 0.773 (0.737-0.805) | 0.533 (0.503-0.563) | 0.480 (0.449-0.512) | 0.807 (0.777-0.835) | 1.654 (1.533-1.785) | 0.467 (0.369-0.494) | 3.542 | 0.306 | -0.496 | 0.184 (0.139-0.229) | <0.001 | 0.119 (0.103-0.135) | <0.001 |
| **Hypertension in the test set** | | | | | | | | | | | | | | | |
| DPN | 0.741 (0.705-0.777) | - | 0.835 (0.784-0.877) | 0.560 (0.513-0.606) | 0.528 (0.480-0.577) | 0.852 (0.805-0.889) | 1.897 (1.688-2.132) | 0.294 (0.224-0.387) | 6.452 | 0.395 | -0.977 | Ref | - | Ref | - |
| ZJU | 0.657 (0.617-0.697) | <0.001 | 0.566 (0.504-0.625) | 0.673 (0.627-0.715) | 0.505 (0.447-0.563) | 0.724 (0.678-0.766) | 1.727 (1.459-2.045) | 0.646 (0.562-0.742) | 2.673 | 0.239 | 42.825 | 0.129 (0.056-0.202) | <0.001 | 0.088 (0.064-0.112) | <0.001 |
| HSI | 0.691 (0.652-0.730) | 0.002 | 0.760 (0.704-0.809) | 0.540 (0.493-0.586) | 0.494 (0.445-0.543) | 0.792 (0.742-0.835) | 1.652 (1.465-1.864) | 0.444 (0.357-0.552) | 3.721 | 0.300 | 35.605 | 0.071 (0.004-0.138) | 0.039 | 0.058 (0.038-0.077) | <0.001 |
| TyG | 0.631 (0.590-0.672) | <0.001 | 0.667 (0.606-0.722) | 0.549 (0.501-0.595) | 0.466 (0.415-0.517) | 0.736 (0.685-0.782) | 1.477 (1.294-1.686) | 0.608 (0.511-0.722) | 2.429 | 0.216 | 9.502 | 0.175 (0.099-0.250) | <0.001 | 0.110 (0.083-0.137) | <0.001 |
| FSI | 0.698 (0.659-0.736) | 0.008 | 0.629 (0.568-0.687) | 0.666 (0.620-0.709) | 0.527 (0.470-0.582) | 0.753 (0.707-0.793) | 1.883 (1.606-2.209) | 0.557 (0.475-0.652) | 3.381 | 0.295 | -0.127 | 0.097 (0.028-0.165) | 0.005 | 0.072 (0.051-0.093) | <0.001 |

Abbreviations: *DPN* diagnostic predictive nomogram, *ZJU* the ZJU index, *HSI* hepatic steatosis index, *TyG* triglycerideglucose index, *FSI* Framingham steatosis index, *AUC* area under the curve, *SEN* sensitivity, *SPE* specificity, *PPV* positive predictive value, *NPV* negative predictive value, *PLR* positive likelihood ratio, *NLR* negative likelihood ratio, *DOR* diagnostic odds ratio, *NRI* net reclassifcation index, *IDI* integrated discrimination improvement

**Table S12** Performance comparison between DPN and other models in predicting MASLD risk among patients without hypertension

| Models | AUC (95% CI) | P1 values | SEN (95% CI) | SPE (95% CI) | PPV (95% CI) | NPV (95% CI) | PLR (95% CI) | NLR (95% CI) | DOR | Yodan | Cut off values | NRI (95% CI) | P2 values | IDI (95% CI) | P3 values |
| --- | --- | --- | --- | --- | --- | --- | --- | --- | --- | --- | --- | --- | --- | --- | --- |
| **Non-hypertension in the training set** | | | | | | | | | | | | | | | |
| DPN | 0.771 (0.748-0.794) | - | 0.791 (0.756-0.822) | 0.624 (0.593-0.655) | 0.567 (0.533-0.601) | 0.827 (0.797-0.853) | 2.104 (1.922-2.304) | 0.335 (0.287-0.392) | 6.281 | 0.415 | -0.779 | Ref | - | Ref | - |
| ZJU | 0.673 (0.646-0.699) | <0.001 | 0.815 (0.782-0.845) | 0.449 (0.417-0.481) | 0.480 (0.449-0.511) | 0.796 (0.759-0.828) | 1.479 (1.382-1.583) | 0.411 (0.347-0.487) | 3.599 | 0.264 | 38.415 | 0.185 (0.132-0.237) | <0.001 | 0.124 (0.107-0.142) | <0.001 |
| HSI | 0.717 (0.692-0.743) | <0.001 | 0.618 (0.578-0.656) | 0.708 (0.679-0.737) | 0.569 (0.530-0.607) | 0.748 (0.719-0.776) | 2.119 (1.886-2.380) | 0.540 (0.487-0.597) | 3.924 | 0.326 | 36.825 | 0.064 (0.017-0.110) | 0.007 | 0.077 (0.061-0.093) | <0.001 |
| TyG | 0.650 (0.623-0.677) | <0.001 | 0.759 (0.723-0.793) | 0.464 (0.432-0.496) | 0.469 (0.437-0.501) | 0.756 (0.719-0.789) | 1.417 (1.317-1.525) | 0.518 (0.449-0.598) | 2.736 | 0.223 | 9.268 | 0.201 (0.148-0.254) | <0.001 | 0.144 (0.125-0.163) | <0.001 |
| FSI | 0.725 (0.700-0.750) | <0.001 | 0.598 (0.558-0.637) | 0.739 (0.710-0.766) | 0.588 (0.548-0.627) | 0.747 (0.718-0.774) | 2.293 (2.025-2.597) | 0.544 (0.493-0.600) | 4.215 | 0.337 | -0.745 | 0.084 (0.038-0.129) | <0.001 | 0.075 (0.060-0.090) | <0.001 |
| **Non-hypertension in the test set** | | | | | | | | | | | | | | | |
| DPN | 0.793 (0.759-0.827) | - | 0.794 (0.739-0.840) | 0.690 (0.643-0.732) | 0.606 (0.552-0.658) | 0.847 (0.805-0.882) | 2.558 (2.195-2.981) | 0.299 (0.235-0.380) | 8.555 | 0.484 | -0.672 | Ref | - | Ref | - |
| ZJU | 0.679 (0.638-0.719) | <0.001 | 0.664 (0.603-0.720) | 0.634 (0.587-0.679) | 0.523 (0.467-0.577) | 0.758 (0.710-0.801) | 1.817 (1.563-2.113) | 0.529 (0.445-0.629) | 3.435 | 0.298 | 40.17 | 0.221 (0.143-0.299) | <0.001 | 0.146 (0.118-0.173) | <0.001 |
| HSI | 0.774 (0.739-0.809) | 0.146 | 0.767 (0.710-0.816) | 0.690 (0.643-0.732) | 0.598 (0.543-0.651) | 0.831 (0.787-0.867) | 2.472 (2.117-2.887) | 0.338 (0.270-0.421) | 7.314 | 0.457 | 35.605 | 0.044 (-0.029-0.116) | 0.240 | 0.043 (0.017-0.069) | 0.001 |
| TyG | 0.637 (0.595-0.679) | <0.001 | 0.718 (0.658-0.770) | 0.510 (0.462-0.558) | 0.469 (0.419-0.519) | 0.750 (0.696-0.797) | 1.465 (1.297-1.656) | 0.553 (0.454-0.674) | 2.649 | 0.228 | 9.281 | 0.269 (0.192-0.346) | <0.001 | 0.179 (0.149-0.209) | <0.001 |
| FSI | 0.768 (0.732-0.808) | 0.045 | 0.702 (0.642-0.756) | 0.715 (0.670-0.756) | 0.597 (0.540-0.652) | 0.799 (0.755-0.837) | 2.464 (2.082-2.916) | 0.416 (0.345-0.502) | 5.923 | 0.417 | -1.011 | 0.060 (-0.008-0.127) | 0.083 | 0.051 (0.029-0.074) | <0.001 |

Abbreviations: *DPN* diagnostic predictive nomogram, *ZJU* the ZJU index, *HSI* hepatic steatosis index, *TyG* triglycerideglucose index, *FSI* Framingham steatosis index, *AUC* area under the curve, *SEN* sensitivity, *SPE* specificity, *PPV* positive predictive value, *NPV* negative predictive value, *PLR* positive likelihood ratio, *NLR* negative likelihood ratio, *DOR* diagnostic odds ratio, *NRI* net reclassifcation index, *IDI* integrated discrimination improvement


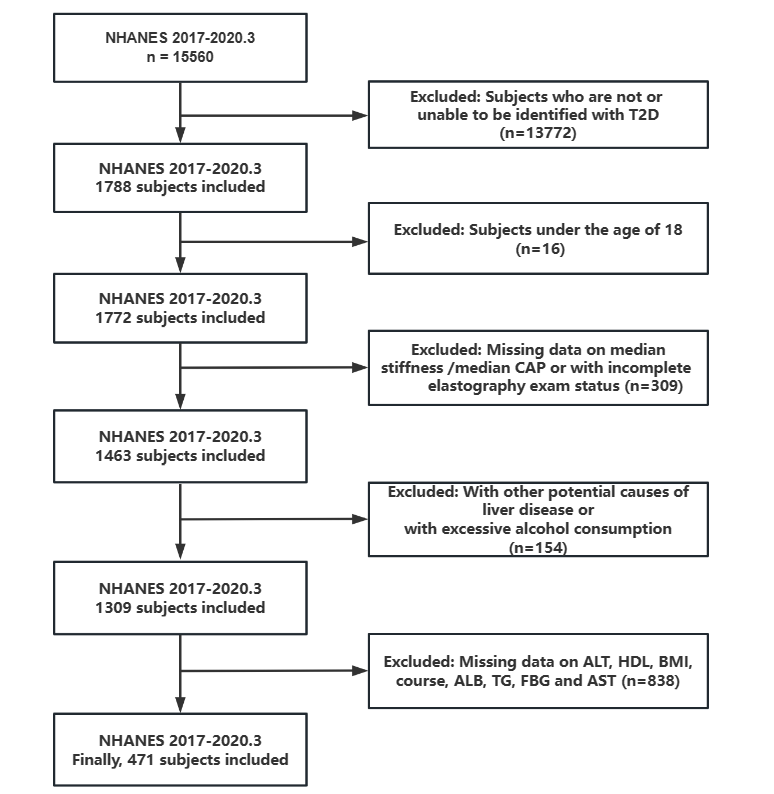


**Fig. S1.** Flowchart of T2DM patients inclusion from the NHANES database


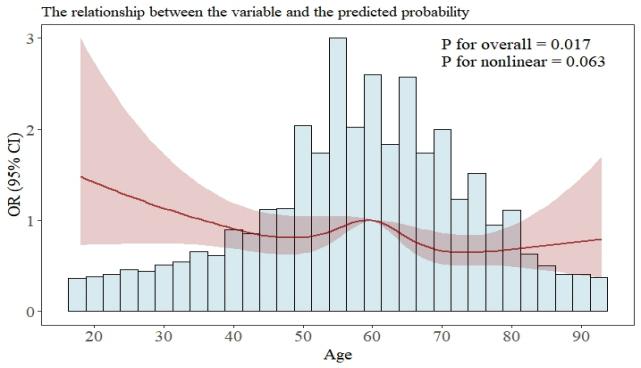

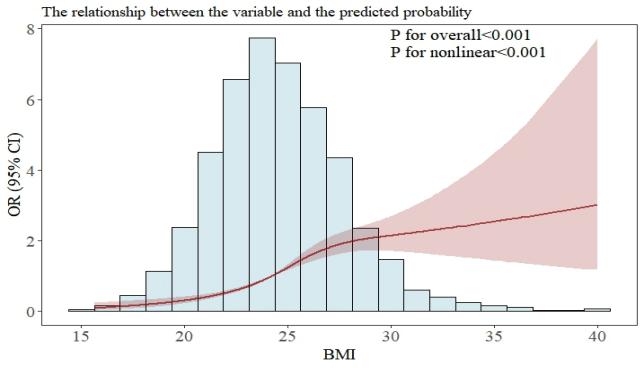

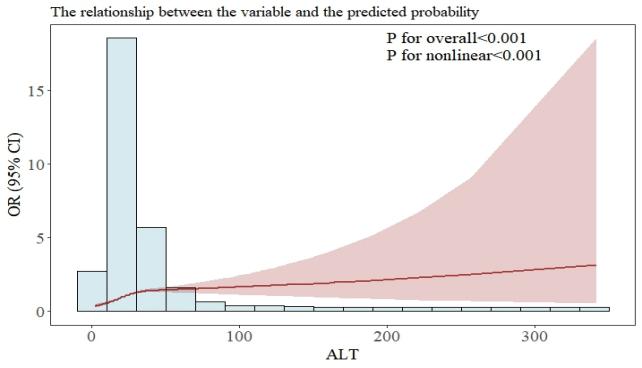

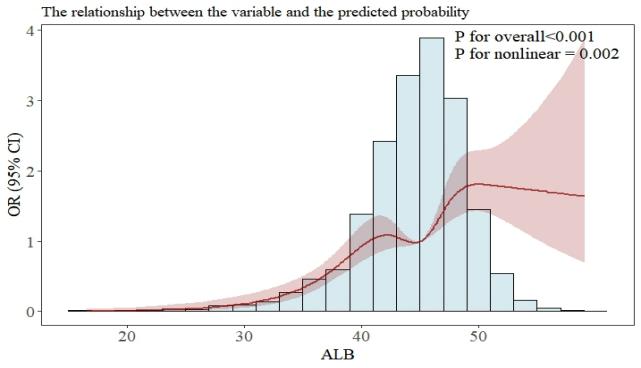

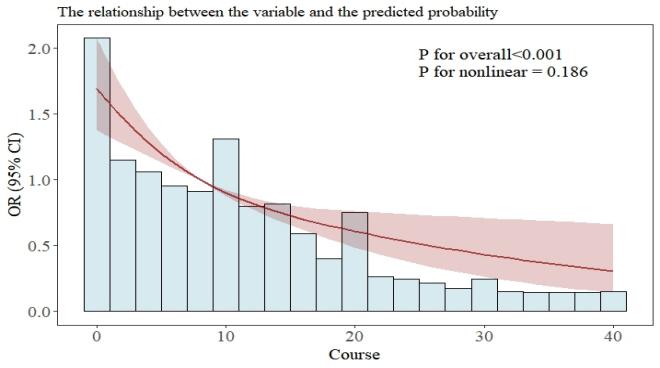

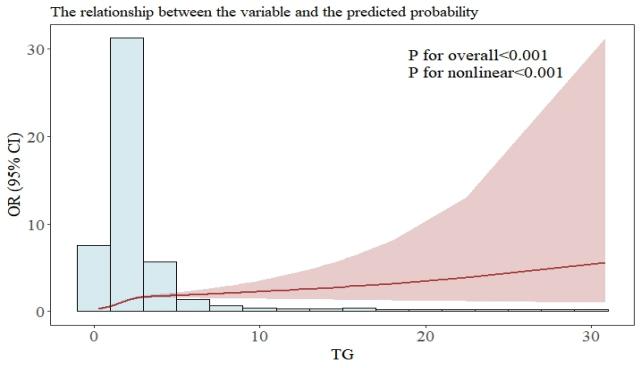

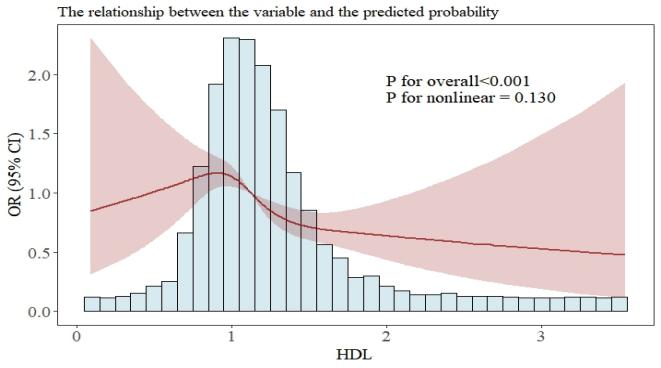


**(A)**

**(B)**

**(C)**

**(D)**

**(E)**

**(F)**

**(G)**

**Fig. S2.** Restricted cubic spline analysis of the relationship between continuous predictive variables and MASLD risk in T2DM patients **(A-G)**.

Abbreviations: *BMI* body mass index, *ALT* alanine transferase, *ALB* albumin, *TG* triglyceride, *HDL-C* high density lipoprotein cholesterol, *OR* odd ratio, *CI* confidence interval.

(**A**)


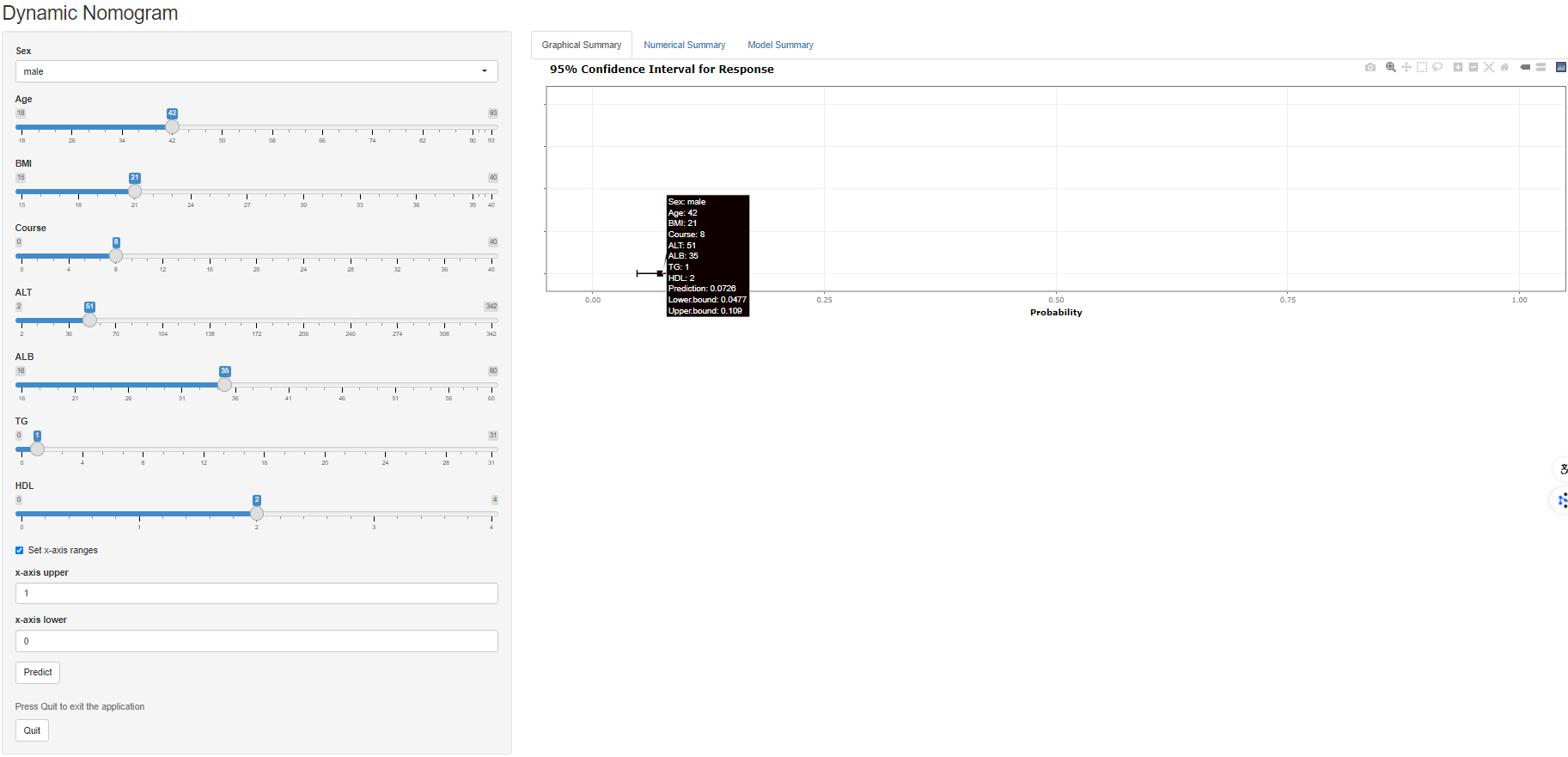


**Fig. S3.** An example risk prediction result (A) and model summary (B)

(**B**)


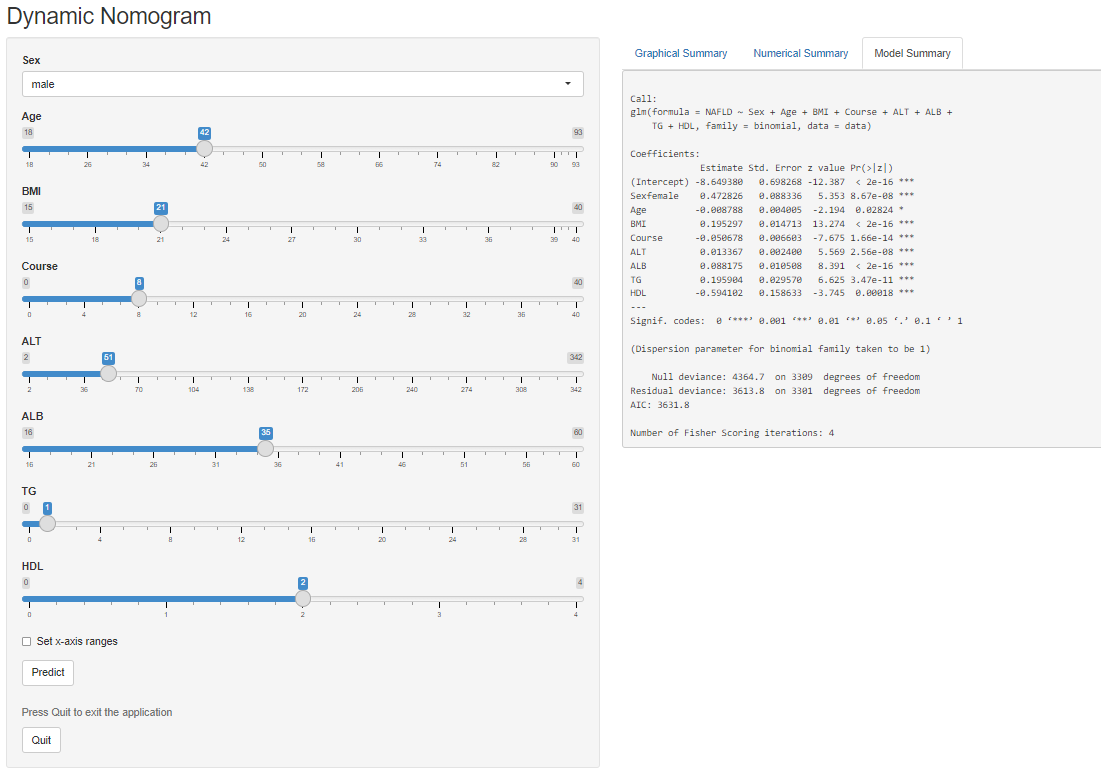


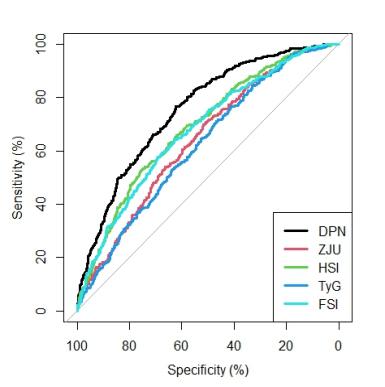

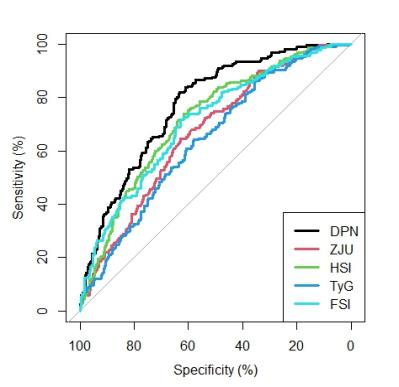

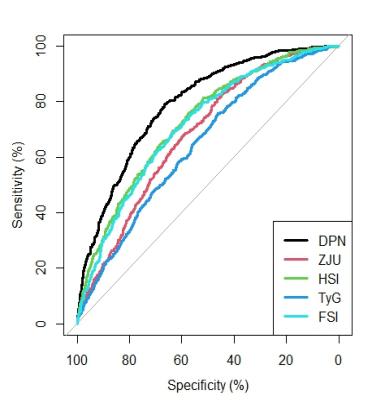

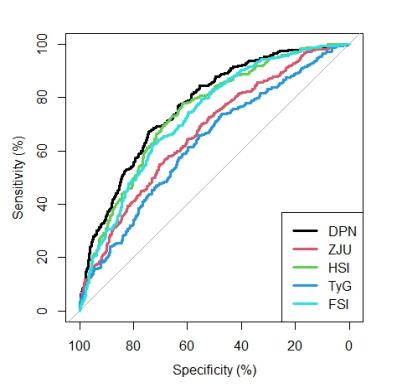

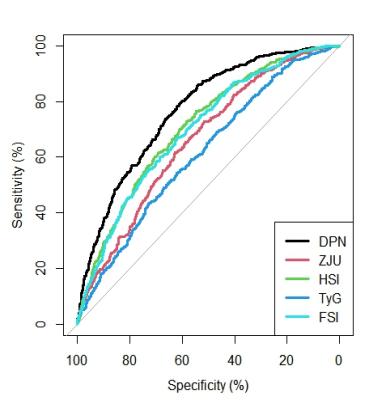

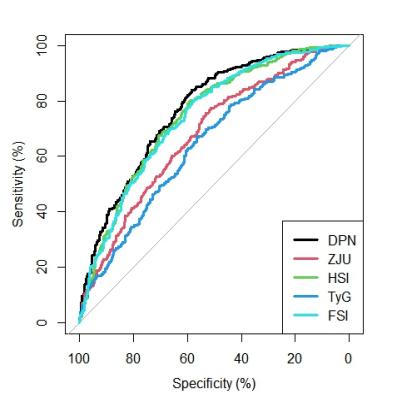

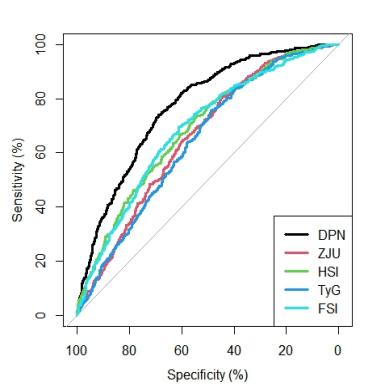

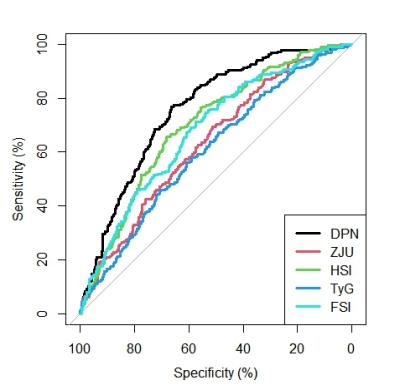

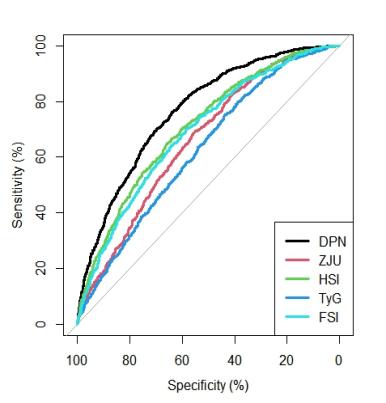

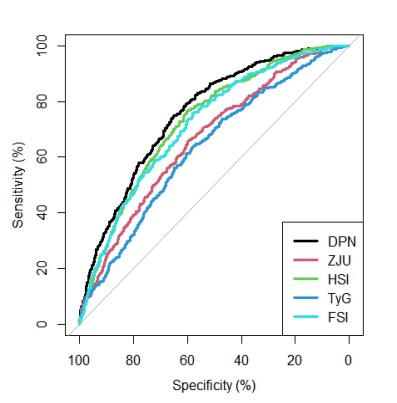

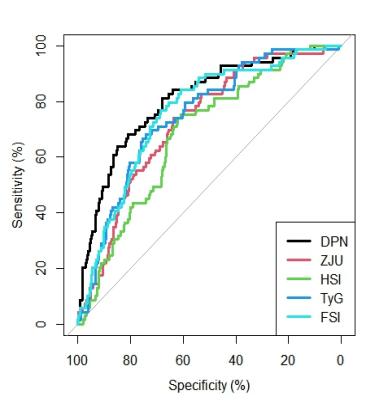

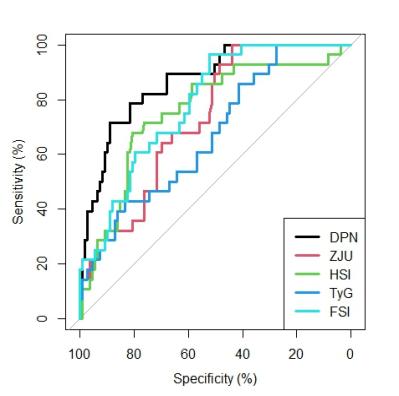

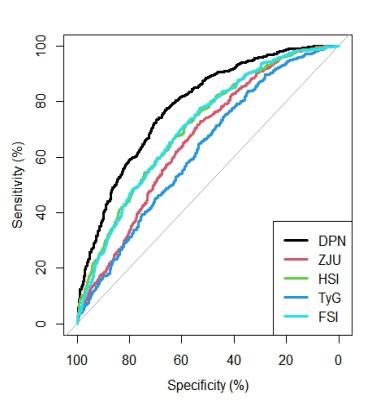

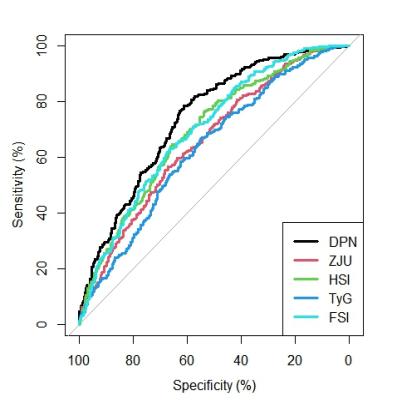

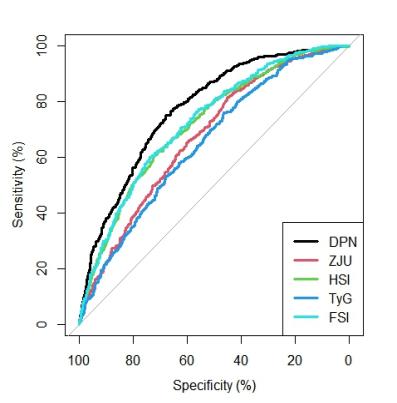

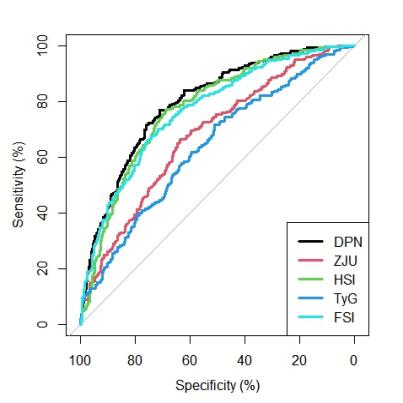


**(A)**

**(B)**

**(C)**

**(D)**

**(E)**

**(F)**

**(G)**

**(H)**

**(I)**

**(J)**

**(L)**

**(K)**

**(O)**

**(M)**

**(P)**

**(N)**

**Fig. S4.** ROC curves of DPN and other models for MASLD prediction across subgroups. (A-B) Male participants in training and validation sets; (C-D) Female participants in training and validation sets; (E-F) Participants aged <60 years in training and validation sets; (G-H) Participants aged ≥60 years in training and validation sets; (I-J) Participants with disease duration <20 years in training and validation sets; (K-L) Participants with disease duration ≥20 years in training and validation sets; (M-N) Hypertensive participants in training and validation sets; (O-P) Non-hypertensive participants in training and validation sets.


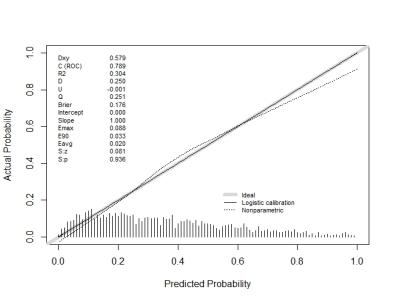

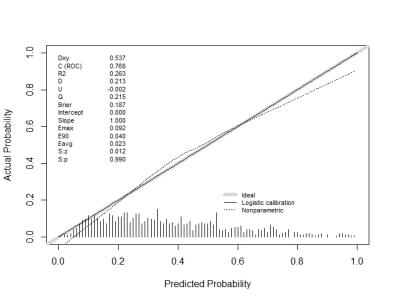

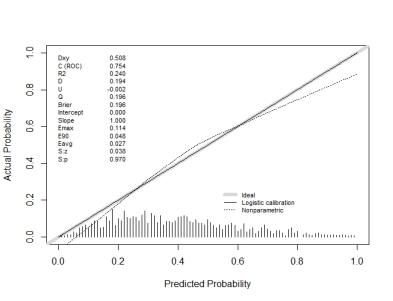

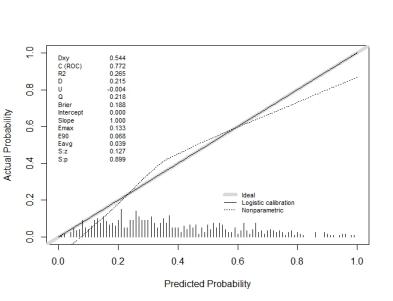

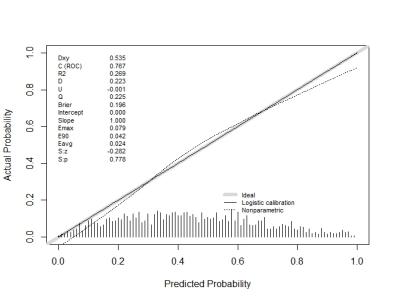

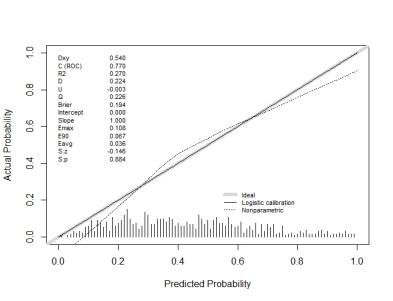

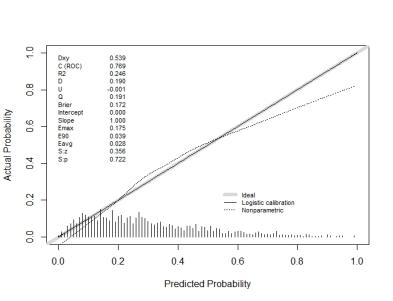

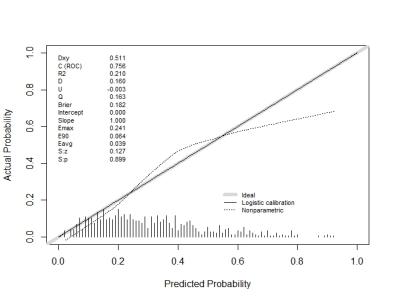

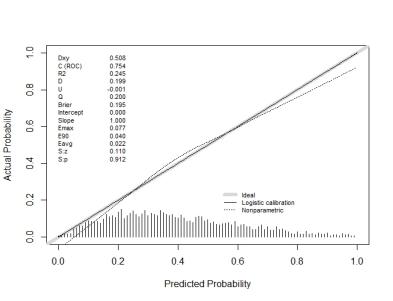

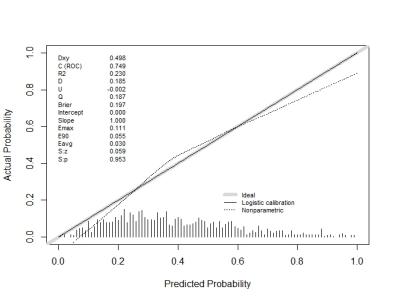

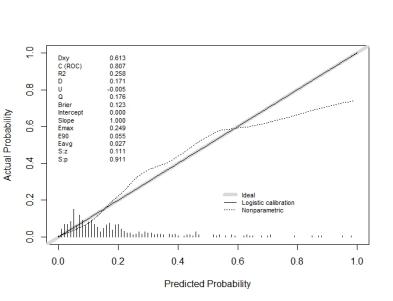

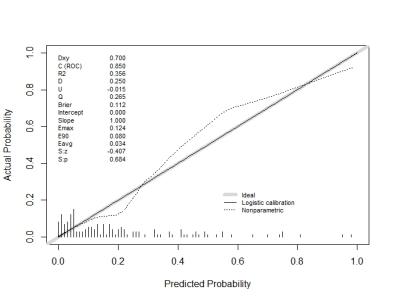

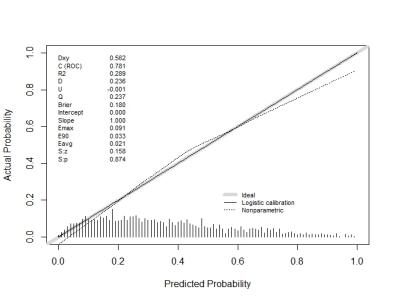

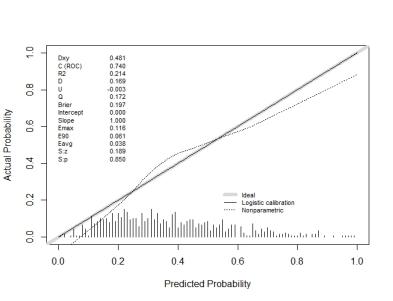

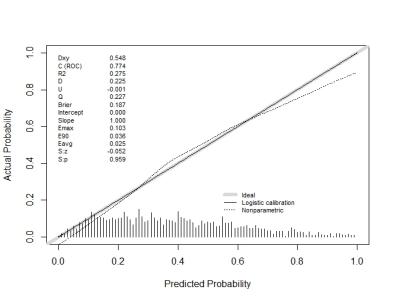

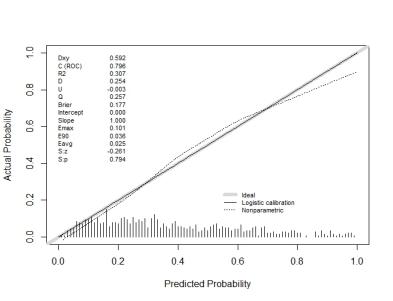


**(A)**

**(K)**

**(J)**

**(I)**

**(H)**

**(G)**

**(F)**

**(E)**

**(D)**

**(C)**

**(B)**

**(P)**

**(O)**

**(N)**

**(M)**

**(L)**

**Fig. S5.** Calibration assessment of DPN and other models for MASLD prediction across subgroups. (A-B) Male participants in training and validation sets; (C-D) Female participants in training and validation sets; (E-F) Participants aged <60 years in training and validation sets; (G-H) Participants aged ≥60 years in training and validation sets; (I-J) Participants with disease duration <20 years in training and validation sets; (K-L) Participants with disease duration ≥20 years in training and validation sets; (M-N) Hypertensive participants in training and validation sets; (O-P) Non-hypertensive participants in training and validation sets.


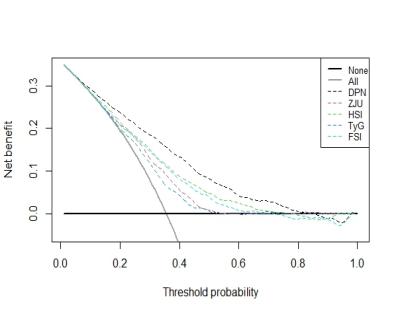

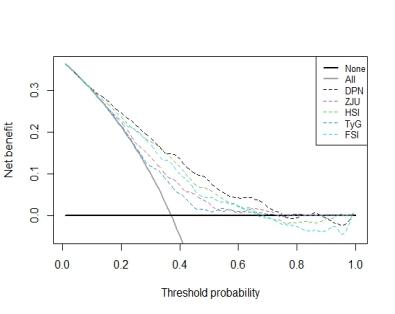

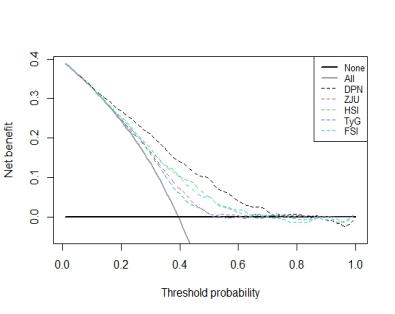

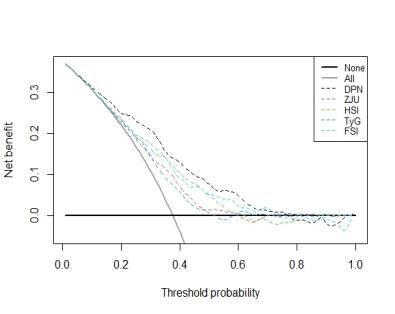

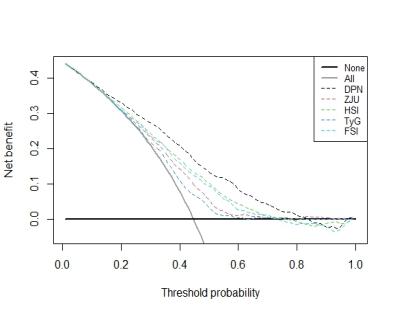

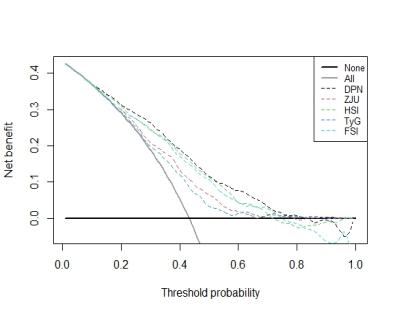

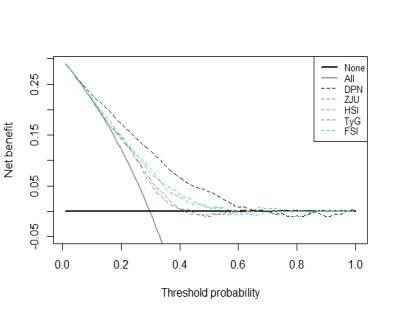

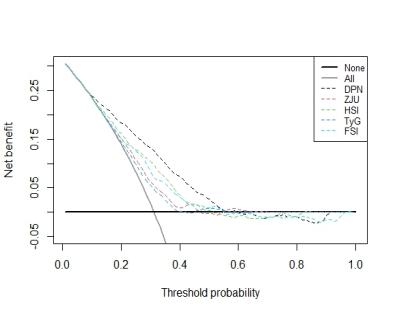

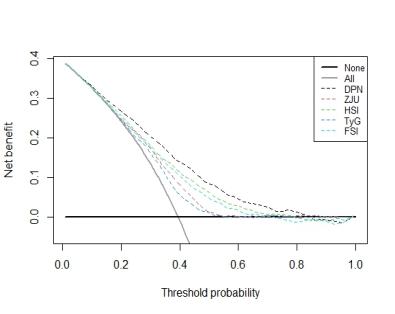

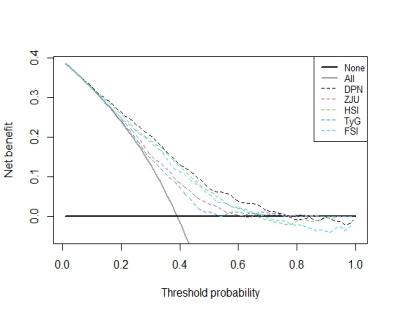

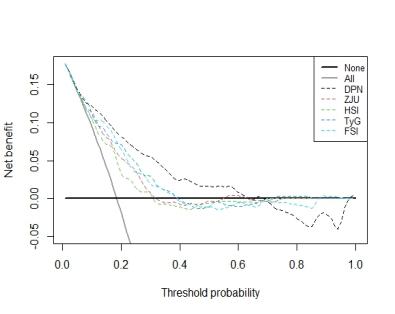

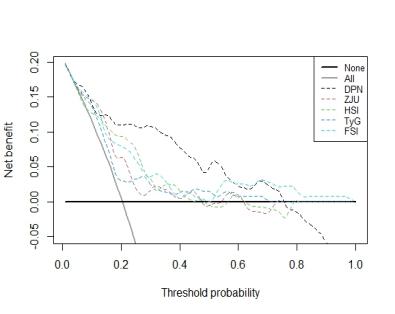

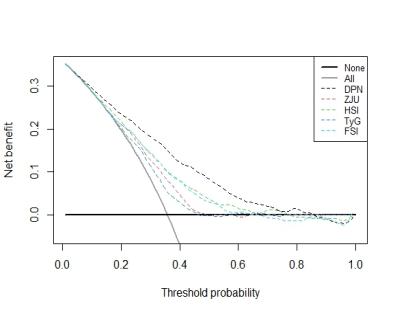

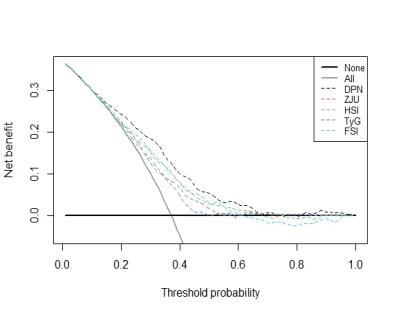

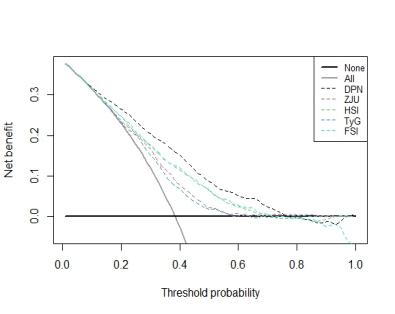

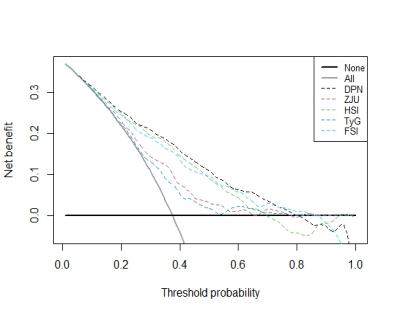


**(A)**

**(P)**

**(O)**

**(N)**

**(M)**

**(L)**

**(K)**

**(J)**

**(I)**

**(H)**

**(G)**

**(F)**

**(E)**

**(D)**

**(C)**

**(B)**

**Fig. S6.** Clinical utility evaluation of DPN and other models across subgroups using DCA. (A-B) Male participants in training and validation sets; (C-D) Female participants in training and validation sets; (E-F) Participants aged <60 years in training and validation sets; (G-H) Participants aged ≥60 years in training and validation sets; (I-J) Participants with disease duration <20 years in training and validation sets; (K-L) Participants with disease duration ≥20 years in training and validation sets; (M-N) Hypertensive participants in training and validation sets; (O-P) Non-hypertensive participants in training and validation sets.


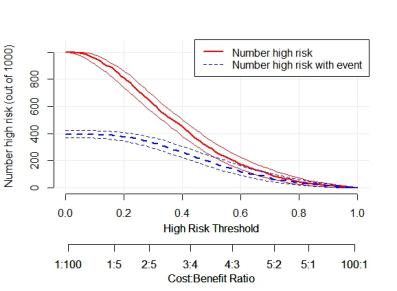

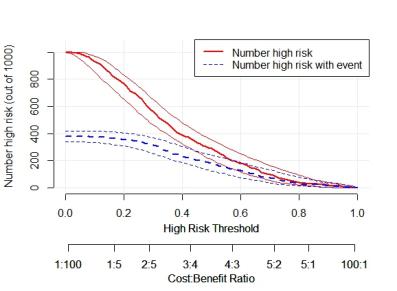

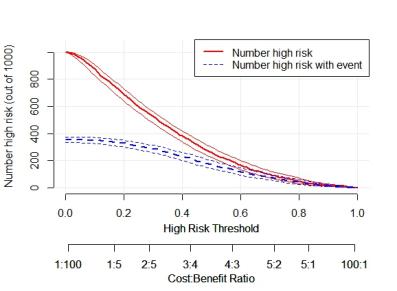

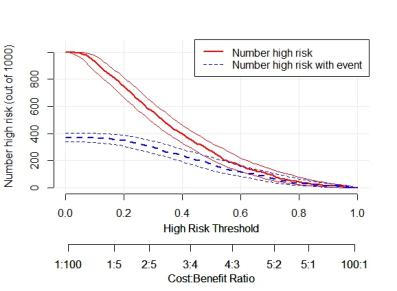

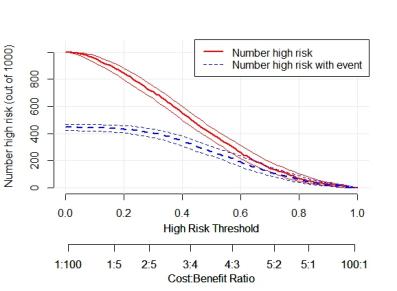

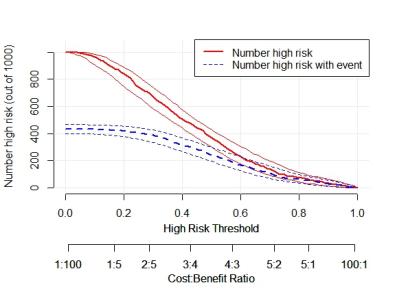

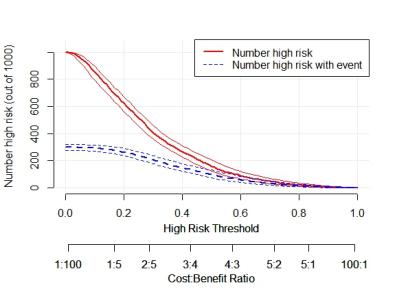

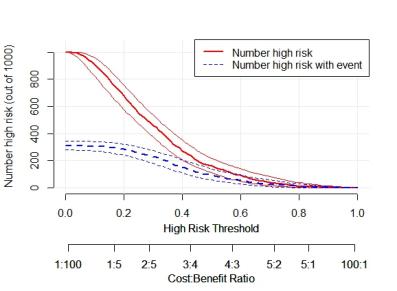

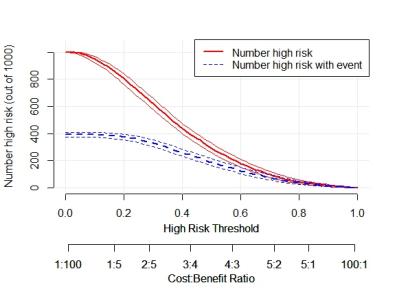

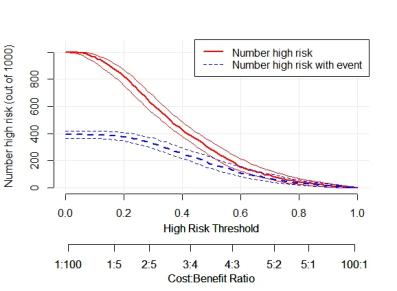

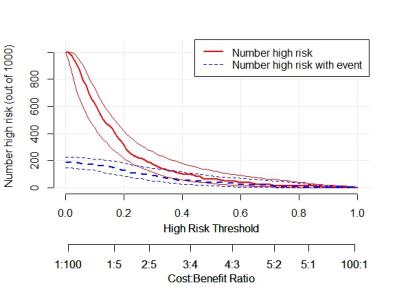

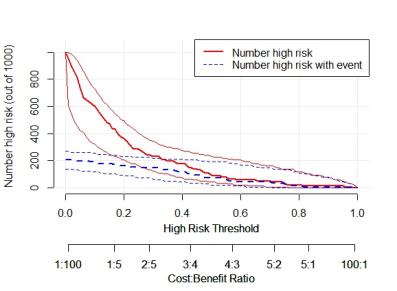

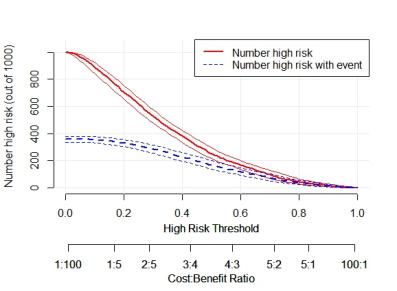

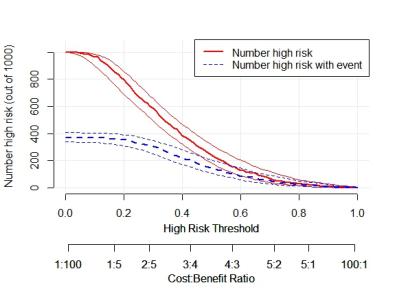

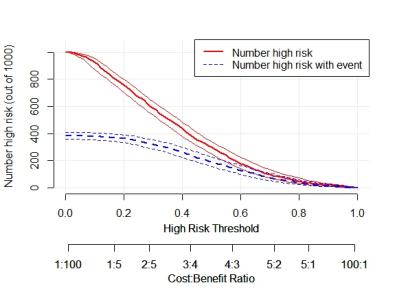

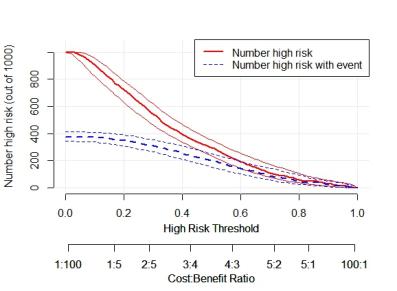


**(A)**

**(P)**

**(O)**

**(N)**

**(M)**

**(L)**

**(K)**

**(J)**

**(I)**

**(H)**

**(G)**

**(F)**

**(E)**

**(D)**

**(C)**

**(B)**

**Fig. S7.** Clinical utility evaluation of DPN and other models across subgroups using CIC. (A-B) Male participants in training and validation sets; (C-D) Female participants in training and validation sets; (E-F) Participants aged <60 years in training and validation sets; (G-H) Participants aged ≥60 years in training and validation sets; (I-J) Participants with diabetes duration <20 years in training and validation sets; (K-L) Participants with diabetes duration ≥20 years in training and validation sets; (M-N) Hypertensive participants in training and validation sets; (O-P) Non-hypertensive participants in training and validation sets.
